# Time-Course Transcriptomic Analysis Identifies *MsTIFY11B* as a Promising Candidate Regulator of Salt–Alkali Tolerance in Alfalfa (*Medicago sativa* L.)

**DOI:** 10.3390/plants15172714

**Published:** 2026-09-04

**Authors:** Linfei Cheng, Yanfeng Liu, Qingchun Liu, Dong Li, Yanan Chen, Jiadong Chen, Yang He, Wei Wang

**Affiliations:** 1 School of Resources and Environmental Engineering, Ludong University, Yantai 264025, China; chenglinfei0902@163.com (L.C.); liuqingchun05@163.com (Q.L.); heyang.waterx@foxmail.com (Y.H.); 2 School of Horticulture, Ludong University, Yantai 264025, China; liuyf3097@126.com (Y.L.); ld@nwafu.edu.cn (D.L.); 15269428077@163.com (Y.C.); chenjiadong1005@163.com (J.C.)

**Keywords:** alfalfa, salt stress, transcriptomics, TIFY family, gene expression

## Abstract

Soil salinization severely limits forage crop productivity, yet the regulatory networks that govern salt stress adaptation in alfalfa, a moderately salt-tolerant leguminous forage, remain largely unexplored. Here, we examined the physiological and transcriptomic dynamics of alfalfa leaves under 200 mM NaCl stress across three time points. Salt stress induced a progressive elevation of the Na^+^/K^+^ ratio, biphasic activation of antioxidant enzymes and concurrent accumulation of malondialdehyde. Time-course RNA-seq analysis identified 3631 differentially expressed genes (DEGs) and 132 core salt-responsive transcription factors (TFs). Pathway and functional annotation analyses indicated that these DEGs were prominently involved in cell wall biogenesis, redox homeostasis, and the carotenoid biosynthesis pathway, with carotenoid accumulation strongly activated under salt stress. Using weighted gene co-expression network analysis (WGCNA), nine distinct co-expression clusters were constructed. Notably, the brown module, which showed a positive correlation with Na^+^ accumulation and the Na^+^/K^+^ ratio, was significantly enriched in the plant hormone signal transduction pathway, within which 72.7% of the enriched genes belonged to the TIFY family. Among them, a core hub gene, *MsTIFY11B*, was isolated for functional characterization. Subcellular localization demonstrated that MsTIFY11B is exclusively localized to the nucleus. Heterologous expression in yeast showed that *MsTIFY11B* overexpression enhanced tolerance to salinity and alkalinity, whereas it conferred negligible protection against mannitol-induced drought stress. Taken together, our findings provide a comprehensive temporal framework of the alfalfa transcriptomic response to salinity and suggest that *MsTIFY11B* may contribute to salt–alkali tolerance, making it a promising candidate for further functional characterization and potential application in the development of stress-adapted alfalfa varieties.

## 1. Introduction

In natural environments, plants encounter diverse abiotic stresses, among which soil salinity represents a major constraint limiting plant growth and agricultural productivity [1]. Currently, salt-affected soils account for approximately 10.7% of global terrestrial areas and 20% of irrigated agricultural lands [2]. More seriously, projections suggest that by 2050, nearly half of agricultural areas may be impacted by salinization driven by climate change and unsustainable irrigation management [3], posing a growing threat to global ecosystems and food security. Consequently, breeding efforts increasingly focus on developing germplasm resources with improved salt tolerance to mitigate the impacts of expanding soil salinity [4]. Such efforts require detailed insights into the physiological and molecular adaptations that govern plant responses to salinity stress.

Salt-induced growth inhibition largely arises from osmotic stress and ionic toxicity within plant cells [5]. High salinity lowers soil water potential, restricting plant water uptake and reducing cell turgor, thereby causing osmotic stress [6]. Elevated Na^+^ and Cl^−^ concentrations interfere with essential nutrient uptake, disturbing intracellular ionic balance and resulting in metabolic disturbances and cellular damage [7]. Additionally, salt stress induces reactive oxygen species (ROS) accumulation, accelerating oxidative processes and causing molecular damage to proteins and cellular membranes [8,9]. To mitigate these adverse effects, plants have evolved diverse adaptive strategies involving morphological, physiological, and biochemical processes [10]. These mechanisms include cell wall remodeling, restoration of intracellular ion homeostasis, biosynthesis of osmoprotectants, optimization of the antioxidant system, and activation of ROS scavenging [11,12]. At the molecular level, plant salt adaptation is governed by a highly coordinated regulatory network integrating stress perception, signaling cascades, ion homeostasis, osmotic adjustment, hormone signaling, and metabolic reprogramming [13,14].

Salt tolerance is a genetically complex trait characterized by significant inter-specific mechanistic divergence and intra-specific adaptive plasticity [15,16]. These strategies, which involve both specialized morphological traits and fundamental physiological reprogramming, are dynamically shaped by genotype, tissue specificity, and stress conditions [17,18]. Therefore, characterizing key genetic regulators and their associated networks is fundamental for developing biotechnology-based strategies to improve plant adaptation to saline environments. In recent years, high-throughput omics and functional genomics technologies have greatly enhanced the discovery and characterization of salt-responsive regulators and molecular networks, providing new insights into the mechanisms underlying salinity adaptation in diverse plant species [19,20,21]. By utilizing high-throughput sequencing, researchers can dynamically monitor the global reprogramming of transcriptomic profiles under salinity stress, facilitating the identification of candidate regulators and their regulatory networks on a genome-wide scale [22,23]. Recent transcriptomic profiling revealed divergent salt adaptation strategies between contrasting cultivars and pinpointed *GmCML48* as a crucial gene contributing to salt tolerance in *Glycine max* [24]. Furthermore, combined transcriptome–metabolome profiling revealed that the phenylpropanoid and flavonoid biosynthesis pathways perform important functions in the response of *Limonium bicolor* to the oxidative and osmotic disturbances induced by salinity [23]. Collectively, transcriptomic profiling is instrumental in deciphering the intricate regulatory landscapes of abiotic stress tolerance and pinpointing genetic targets for developing superior salt-tolerant germplasm.

Alfalfa (*Medicago sativa* L.) is a widely cultivated forage legume valued for its high biomass productivity and superior forage quality [25]. It serves not only as a cornerstone forage resource for the livestock industry but also contributes substantially to agroecosystem sustainability, owing to its efficient nitrogen fixation capacity and extensive root system [26]. Despite its moderate inherent salinity tolerance, soil salinization remains a primary abiotic constraint that compromises both biomass yield and forage quality by impairing seed germination, stunting root architecture, and reducing photosynthetic capacity [5,27]. With increasing global land salinization driven by climatic shifts and unsustainable land use, the generation of salt-adapted cultivars has become an urgent priority for maintaining stable livestock production and improving the utilization of marginal saline-alkali lands [28,29]. Although multi-omics studies have provided valuable insights into alfalfa responses to salinity and revealed several stress-associated genes, the contributions of additional regulatory components and their precise molecular mechanisms remain largely elusive [29,30,31,32]. Consequently, leveraging transcriptomics to identify novel salt-responsive candidate genes and elucidate their underlying pathways is essential for refining the regulatory network of alfalfa.

In the present study, we combined physicochemical characterization with transcriptome-wide analysis to elucidate the regulatory processes governing alfalfa adaptation to salt stress. Our primary objective was to characterize global expression patterns and identify key DEGs and pathways associated with salinity tolerance. By correlating physiological shifts with transcriptomic profiles, we identified candidate genes and regulatory networks associated with salt tolerance, providing a basis for subsequent functional validation and elucidation of the underlying regulatory mechanisms governing salt–alkali stress responses.

## 2. Results

### 2.1. Physiological and Biochemical Responses of Alfalfa to Salt Stress

**Figure 1:**
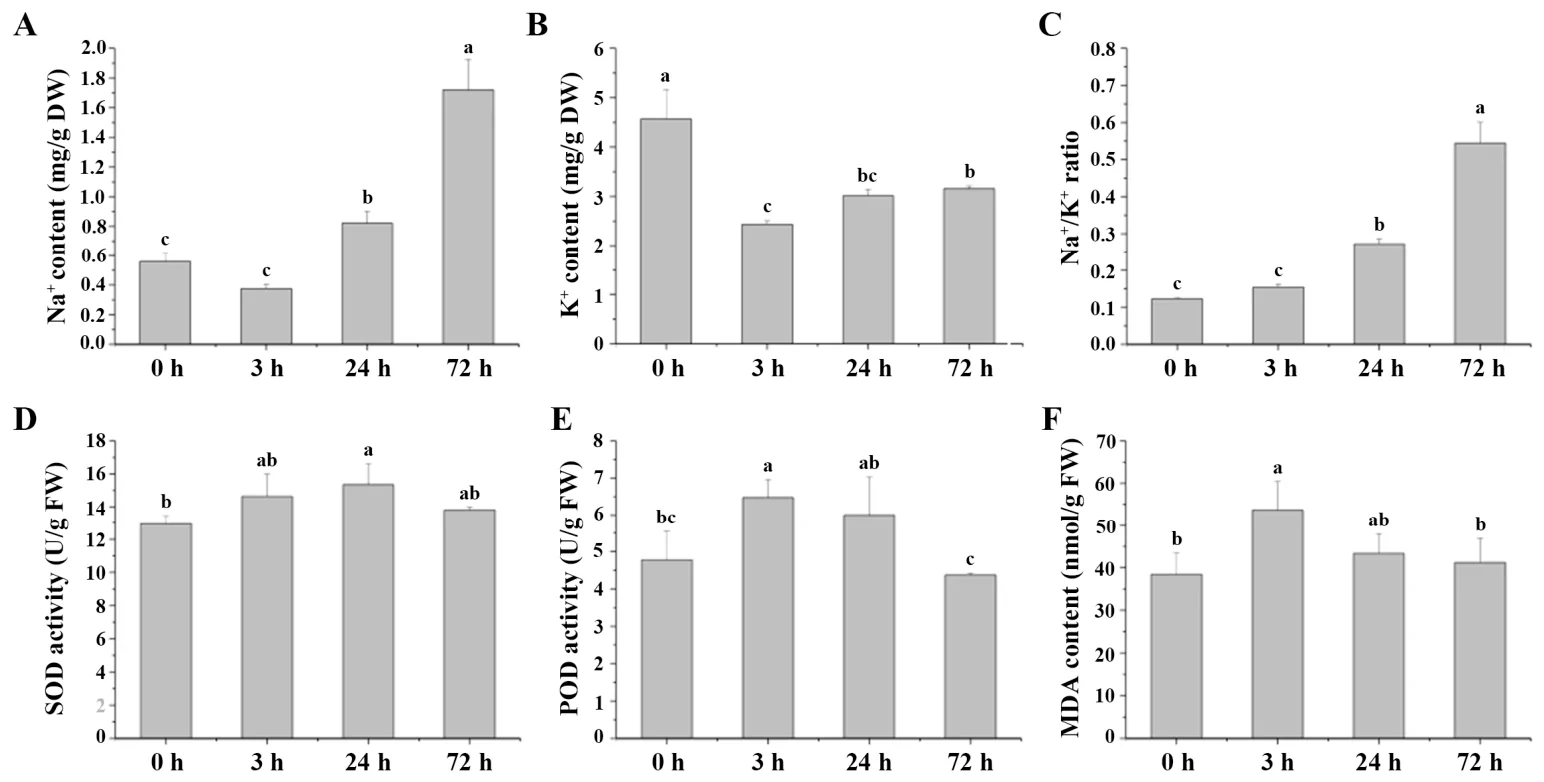
Physiological and biochemical responses of alfalfa under salt stress. (**A**) Na^+^ content. (**B**) K^+^ content. (**C**) Na^+^/K^+^ ratio. (**D**) SOD activity. (**E**) POD activity. (**F**) MDA content. Data are presented as means ± SD of three independent biological replicates. Statistical significance was determined by one-way ANOVA followed by Fisher’s LSD post hoc test. Different lowercase letters indicate statistically significant differences between groups (*p* < 0.05).

In this study, we characterized the dynamic changes in the physiological and biochemical parameters of alfalfa under salt stress. Overall, leaf Na^+^ concentrations increased, whereas K^+^ levels declined under salt treatment compared with those in untreated plants, resulting in an elevated Na^+^/K^+^ ratio (Figure 1A–C). Additionally, the activities of superoxide dismutase (SOD) and peroxidase (POD), along with malondialdehyde (MDA) accumulation, exhibited a transient increase followed by a gradual decline over the 72-h stress period (Figure 1D–F). Specifically, SOD activity peaked at 24 h, whereas POD activity reached its maximum at 3 h. MDA content rose sharply during the initial 3 h and then gradually decreased, which may indicate an early stress response, followed by possible physiological adjustment at 24 h and partial recovery by 72 h.

### 2.2. Temporal Transcriptomic Dynamics in Alfalfa Leaves Following Salt Treatment

To dissect the transcriptional responses of alfalfa to salt stress, transcriptome sequencing was performed using leaf samples. Sequencing of the twelve libraries generated approximately 41.84–47.10 million raw reads, with 41.76–47.00 million clean reads retained after quality filtering (Table S1). The proportion of clean reads exceeded 99%, and 34,950 expressed genes were identified across all samples. The principal component analysis (PCA) performed on transcriptome profiles using FPKM values showed that PC1 and PC2 accounted for 28.42% and 22.70% of the variance, respectively (Supplementary Figure S1A). Pearson’s correlation analysis of transcriptome samples demonstrated strong correlations (*R*^2^ > 0.9) between biological replicates under the same treatment conditions, indicating high reproducibility of the RNA-seq datasets (Supplementary Figure S1B).

**Figure 2:**
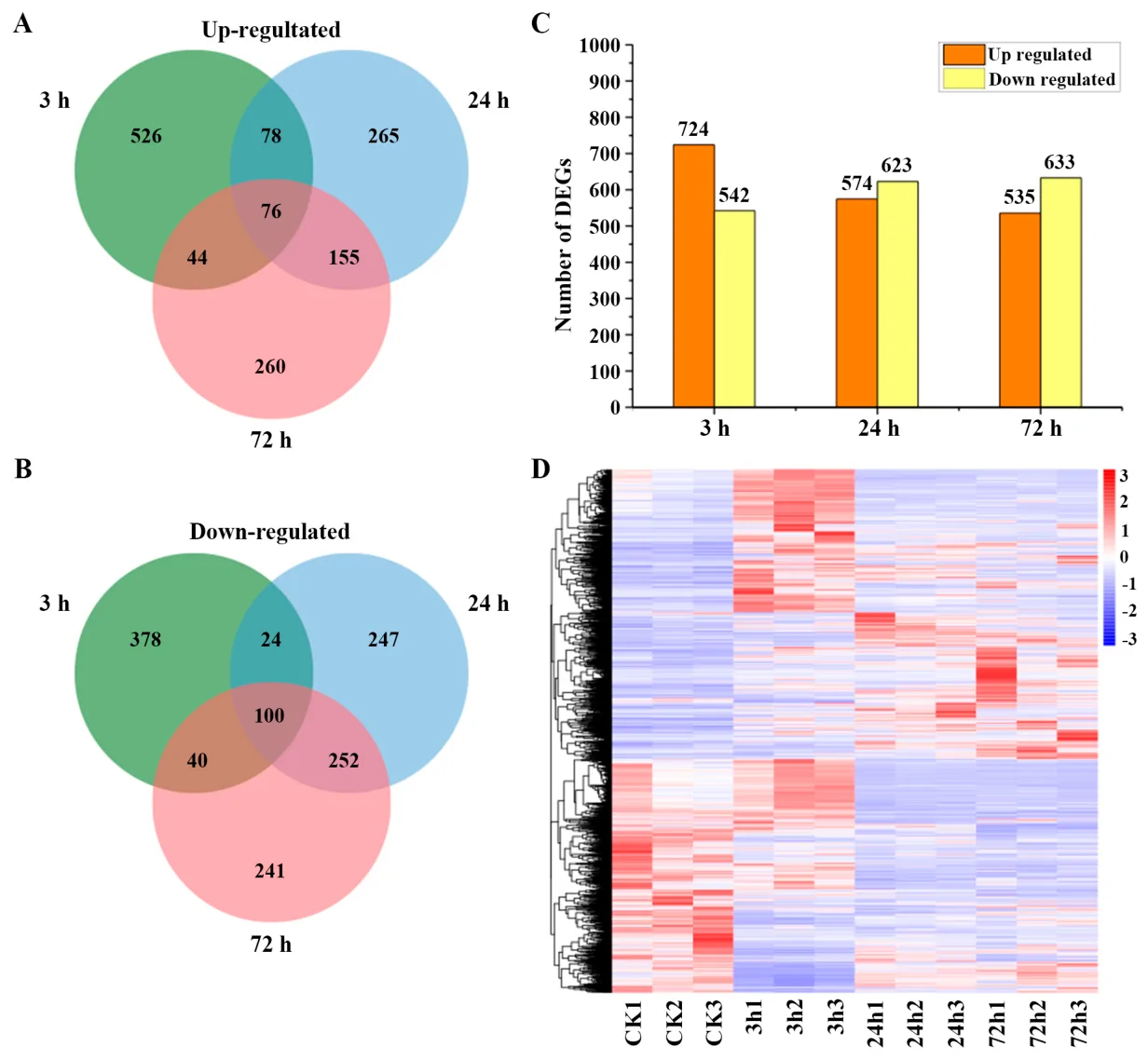
Overview of salt stress-responsive DEGs. (**A**,**B**) Venn diagram illustrating the overlap among up-regulated and down-regulated genes, respectively. (**C**) Number of DEGs at different time points. (**D**) Heatmap showing the expression profiles of DEGs across the three time points. CK represents the control group; 3 h, 24 h, and 72 h indicate samples collected at 3, 24, and 72 h after salt stress treatment, respectively.

In comparison with non-treated control plants, 3631 genes were identified as DEGs under salt stress conditions (Figure 2A–C; Table S2). Among them, 526 and 378 genes were specifically up- and down-regulated at 3 h, 265 and 247 at 24 h, and 260 and 241 at 72 h, respectively. The Venn diagram showed that 76 up-regulated and 100 down-regulated genes were commonly detected across the three time points (Figure 2A,B), indicating that shared DEGs represented only a small proportion of all identified DEGs, whereas most genes exhibited time-point-specific expression patterns. Furthermore, hierarchical clustering of all DEGs revealed that a subset of genes exhibited marked differences in their expression profiles among the experimental groups (Figure 2D). To validate the RNA-seq expression patterns, nine salt-induced DEGs were examined using RT-qPCR (Supplementary Figure S2A–I). Our findings showed a strong correlation with the RNA-seq data (*R*^2^ = 0.89), supporting the reliability of the transcriptomic findings (Supplementary Figure S2J).

### 2.3. GO and KEGG Enrichment Analysis of DEGs

**Figure 3:**
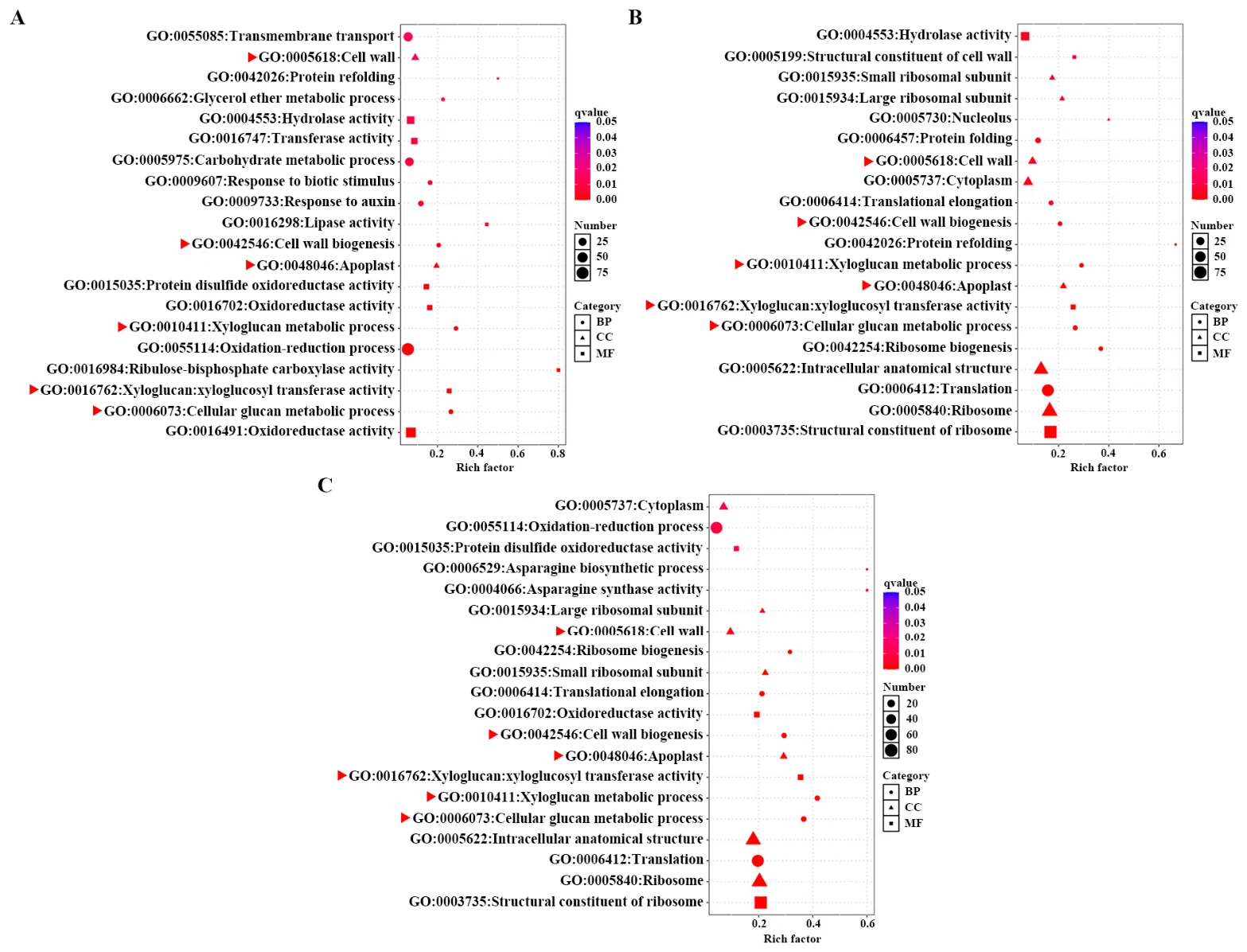
GO analyses of salt stress-responsive DEGs. (**A**–**C**) Top 20 significantly enriched GO terms at different time points after salt treatment. The triangle to the left of each subfigure indicates GO terms that were significantly enriched at all time points following salt treatment.

To characterize the functional implications of salt-responsive DEGs in alfalfa, we performed Gene Ontology (GO) and Kyoto Encyclopedia of Genes and Genomes (KEGG) pathway analyses of these genes. After 3, 24, and 72 h of salt treatment, a total of 1266, 1197, and 1168 DEGs were enriched and classified into 29, 36, and 30 GO terms, respectively (Table S3). After removing duplicates, 60 enriched GO terms were identified across all time points, comprising 26 biological process (BP), 10 cellular component (CC), and 24 molecular function (MF) terms. Across the three time points, salt-induced DEGs were commonly enriched in several GO categories, including xyloglucan metabolic process (GO:0010411, BP), cell wall biogenesis (GO:0042546, BP), cell wall (GO:0005618, CC), protein disulfide oxidoreductase activity (GO:0015035, MF), and hydrolase activity (GO:0004553, MF) (Figure 3A–C; Table S3). Additionally, the oxidation-reduction process (GO:0055114, BP) and oxidoreductase activity (GO:0016702, MF) were consistently and significantly enriched at both 3 and 72 h after salt stress exposure. Together, these findings indicate that the identified DEGs in alfalfa leaves respond to salt stress primarily through involvement in the above enriched terms, with particularly prominent roles in cell wall-related processes and oxidation-reduction reactions. This implies that genes associated with these categories are likely to participate in the regulatory processes underlying salt adaptation in alfalfa.

**Figure 4:**
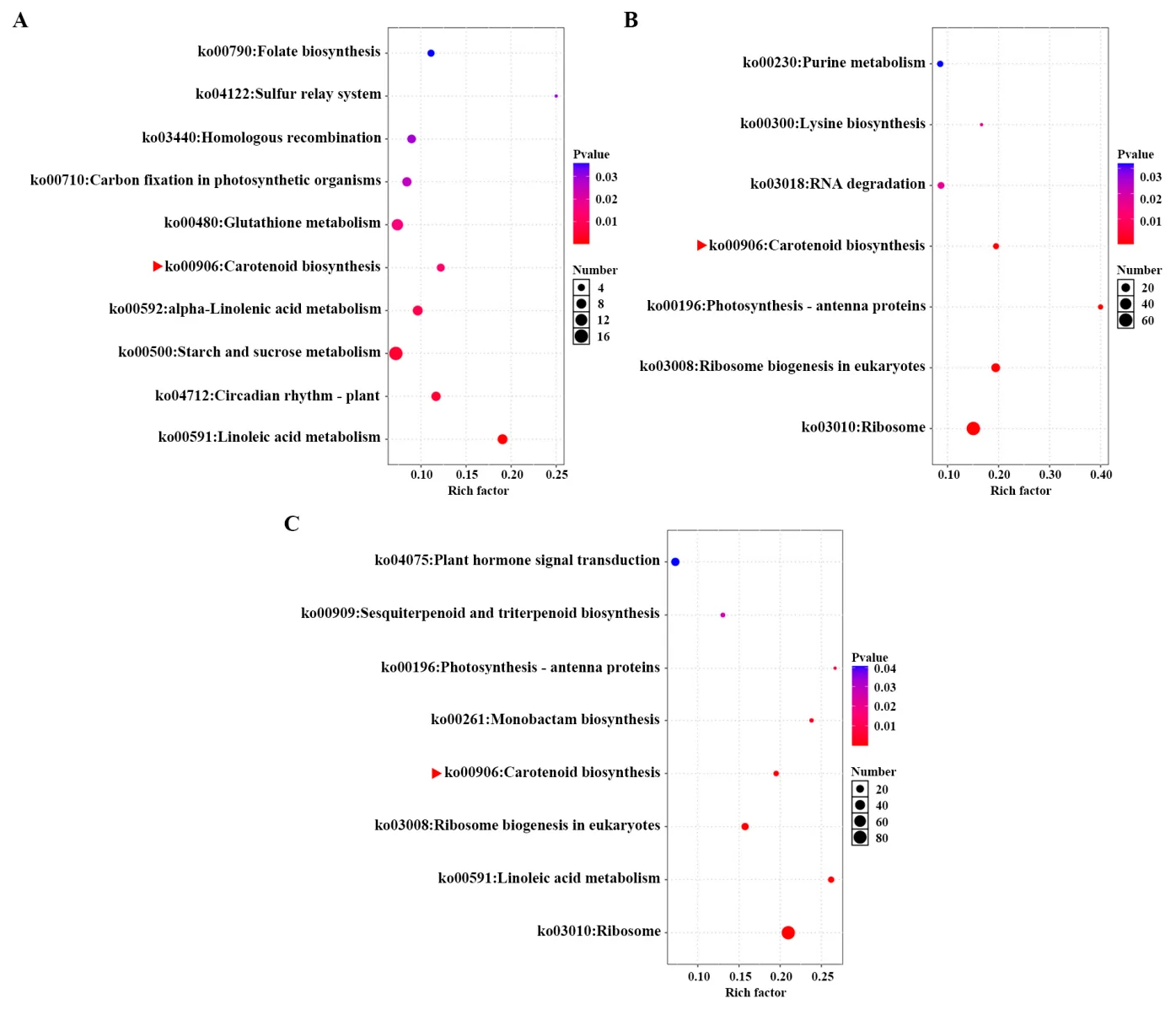
KEGG analyses of salt stress-responsive DEGs. (**A**–**C**) Significantly enriched KEGG pathways at different time points after salt treatment. The triangle to the left of each subfigure indicates a KEGG pathway that was significantly enriched at all time points following salt treatment.

Subsequently, KEGG enrichment analysis revealed that 10, 7, and 8 pathways were significantly enriched under 3, 24, and 72 h salt treatments, respectively (Figure 4A–C; Table S4). Among these, carotenoid biosynthesis (ko00906), a pathway belonging to terpenoids and polyketides metabolism, was consistently enriched across all time points. Furthermore, photosynthesis-related pathways displayed stage-specific enrichment patterns: carbon fixation in photosynthetic organisms (ko00710) was specifically enriched at 3 h, whereas photosynthesis–antenna proteins (ko00196) became more prominent at 24 and 72 h (Figure 4A–C).

To explore the association between carotenoid accumulation and salinity adaptation in alfalfa, we quantified the dynamic changes in chlorophyll and carotenoid content during the salt stress period. The results showed that chlorophyll content decreased slightly with prolonged salt stress, whereas carotenoid content increased significantly under salt treatment (Supplementary Figure S3). These findings suggest that carotenoid accumulation likely contributes to the adaptive responses of alfalfa to salinity stress.

### 2.4. Identification of Salt-Responsive Transcription Factors (TFs) in Alfalfa

**Figure 5:**
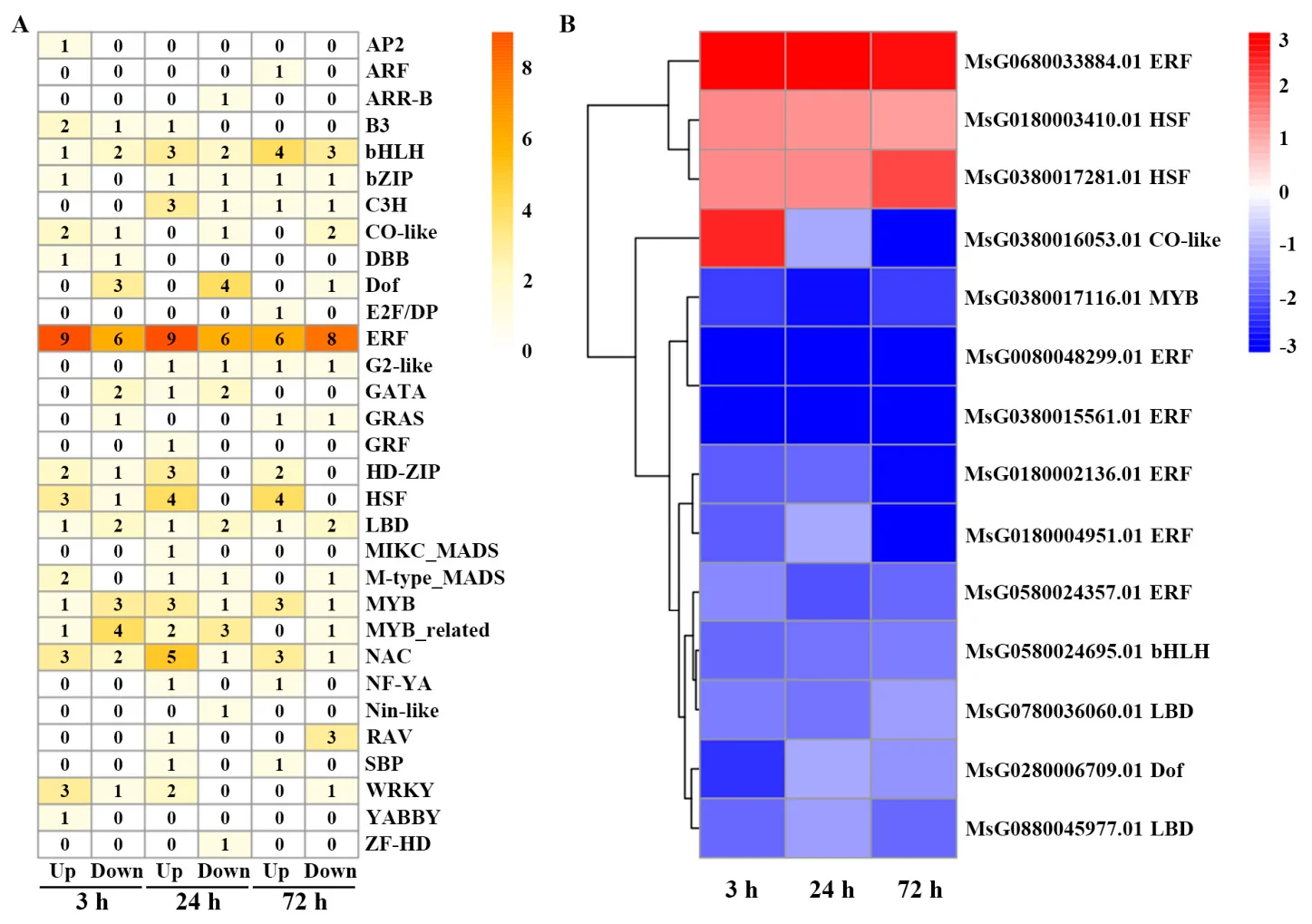
Distribution and clustering of salt stress-responsive differentially expressed transcription factors (TFs). (**A**) Distribution of up-regulated (Up) and down-regulated (Down) differentially expressed TFs at different time points after salt treatment. The color scale indicates the number of differentially expressed TFs. (**B**) Clustering heatmap showing the expression profiles of overlapping differentially expressed TFs across the three time points.

To investigate the contribution of TFs to salt adaptation in alfalfa and uncover their potential regulatory functions, salt-responsive TF genes were screened and characterized. In total, 132 non-redundant genes from 31 TF families showed significant transcriptional changes under salt stress conditions (Figure 5; Table S5). Across all time points, up-regulated transcription factors consistently outnumbered down-regulated ones, increasing from 58.5% at 3 h to 63.2% at 24 h, then to 60.5% at 72 h. Down-regulated TFs accounted for the remaining 41.5%, 36.8%, and 39.5%, respectively. Several TF families, including ERF, bHLH, and NAC, were recurrently regulated, whereas some families like AP2, ARF, GRF, and Nin-like were detected only at a single time point (Figure 5A). Additionally, 14 TFs exhibited consistent expression changes across the three time points. Notably, 10 TF genes, including 5 ERF, 2 LBD, 1 MYB, 1 bHLH, and 1 Dof, were stably down-regulated throughout the stress period. In contrast, one ERF and two HSF genes were persistently up-regulated across time points. A CO-like transcription factor displayed distinct temporal expression patterns, being up-regulated at 3 h but down-regulated at 24 h and 72 h (Figure 5B). These results revealed that salt stress induces dynamic and time-specific changes in the expression of numerous transcription factor families, which may coordinate distinct transcriptional programs at different phases of the stress response.

### 2.5. Characterization of WGCNA Modules Linked to Phenotypic Traits

**Figure 6:**
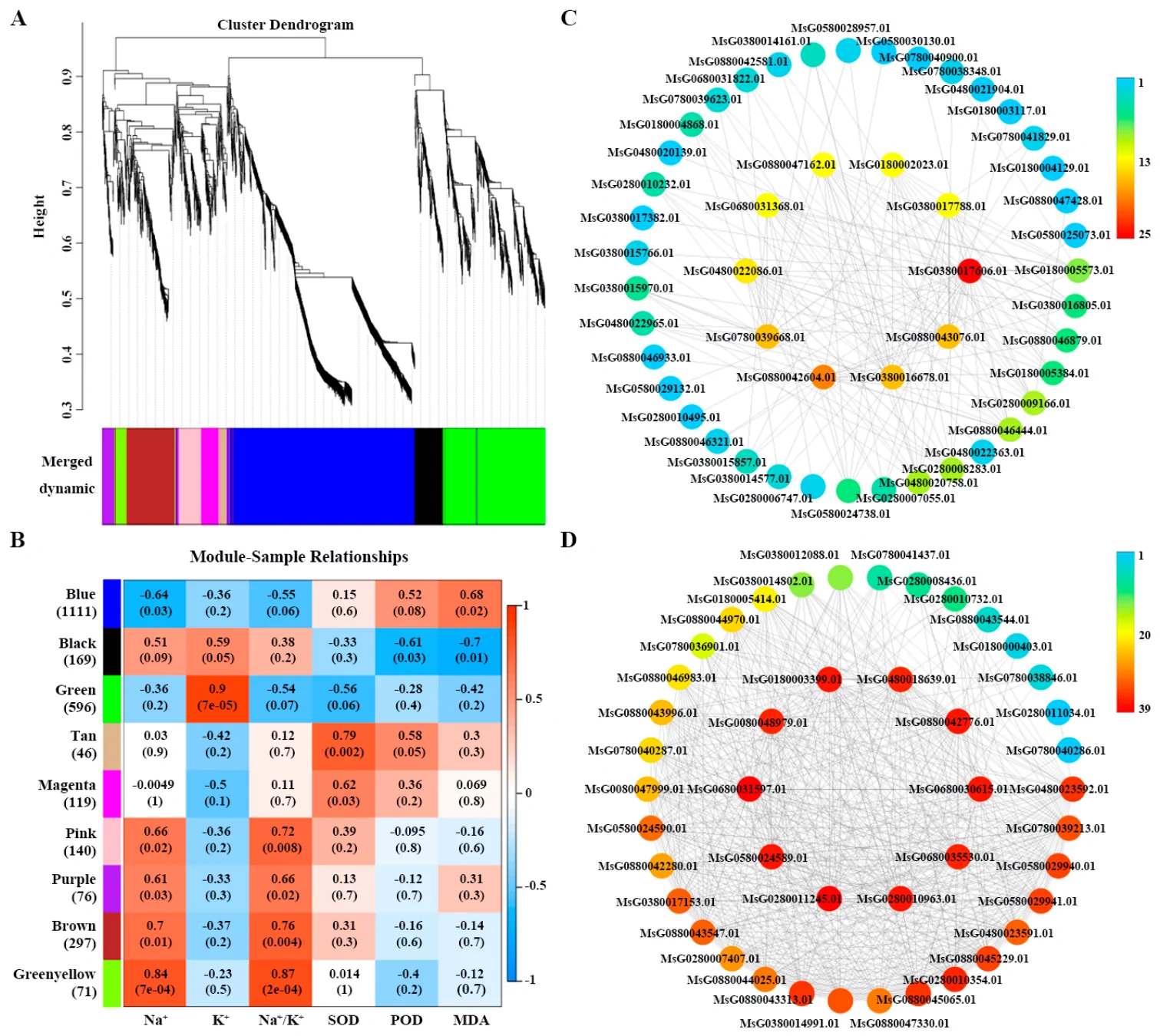
WGCNA of salt-responsive genes in alfalfa. (**A**) Gene clustering dendrogram and module identification. Each branch represents a gene and different modules were assigned distinct colors. (**B**) Module–trait relationship heatmap. Rows represent individual modules, with cells displaying the correlation coefficients and *p*-values between module eigengenes and the associated traits. (**C**,**D**) Co-expression networks in the blue and brown modules. Node color intensity reflects gene connectivity.

To explore the regulatory network under salt stress, a WGCNA was performed to detect trait-linked modules using the expression profiles of 2625 non-redundant DEGs (Table S6). A total of nine co-expression modules, comprising 46 to 1111 genes, were identified by WGCNA using a soft-threshold power of 7 (Figure 6A,B; Table S6). Evaluation of module–trait associations revealed that different modules correlated distinctly with different traits. For example, the black module was significantly negatively correlated with POD activity and MDA content, while the tan module showed a significant positive correlation with SOD activity. Additionally, four modules, including pink, purple, brown, and green-yellow, were positively associated with Na^+^ content and Na^+^/K^+^ ratio (Figure 6B). These findings suggest that a complex regulatory network governs salt-stress response in alfalfa, wherein different genes likely operate through diverse pathways to execute their functions.

To characterize the functional features of individual modules, KEGG enrichment analysis was performed for all modules. Analysis showed that the blue module, comprising 1111 genes, exhibited a significant negative correlation with Na^+^ content and a positive association with MDA content, while being significantly enriched in the ribosome (ko03010), ribosome biogenesis in eukaryotes (ko03008), and Circadian rhythm-plant (ko04712) (Table S7). The brown module, which contained 297 genes showing significant positive correlations with both Na^+^ content and Na^+^/K^+^ ratio, was significantly enriched in linoleic acid metabolism (ko00591), terpenoid backbone biosynthesis (ko00900), and plant hormone signal transduction (ko04075) (Table S7). In contrast, genes in the other seven modules were not enriched in any biological pathways. Therefore, based on the calculated weight values, we constructed module-specific co-expression networks for the blue and brown modules using a threshold of edge weight > 0.65 and >0.50, respectively (Figure 6C,D). The blue module-associated co-expression network contained 49 genes, 51.0% (25) of which were annotated as ribosomal proteins (Figure 6C; Table S8). In contrast, the brown module network contained 44 genes with more diverse functional annotations, such as Cytochrome P450, AP2/ERF, TIFY, and Bet v I/Major latex protein (Figure 6D; Table S8).

### 2.6. Subcellular Localization and Functional Analysis of MsTIFY11B in Yeast

Expression profile analysis revealed that blue module genes were generally down-regulated under salt stress, whereas genes assigned to the brown module showed predominantly up-regulated expression. KEGG enrichment analysis revealed that genes in the brown module were primarily involved in the plant hormone signal transduction pathway (ko04075) (Table S7). Notably, among the 11 genes assigned to this pathway, 8 were assigned to the TIFY family. TIFY proteins are plant-specific regulators that are indispensable for jasmonate (JA) signaling, a pathway recognized as an important regulator of plant adaptation to adverse environmental conditions. These findings suggest that these candidate TIFY genes likely contribute to salt adaptation in alfalfa. Therefore, *MsG0880042776.01*, which exhibited the highest degree value among all TIFY family members in the brown module, was identified as a core TIFY hub gene and selected for functional validation (Table S8). Phylogenetic analysis revealed that MsG0880042776.01 was most closely related to *Arabidopsis thaliana* TIFY11B, sharing 35% protein sequence identity, and was accordingly designated as *MsTIFY11B* (Supplementary Figure S4). We subsequently characterized the function of *MsTIFY11B* to elucidate its underlying molecular mechanisms.

**Figure 7:**
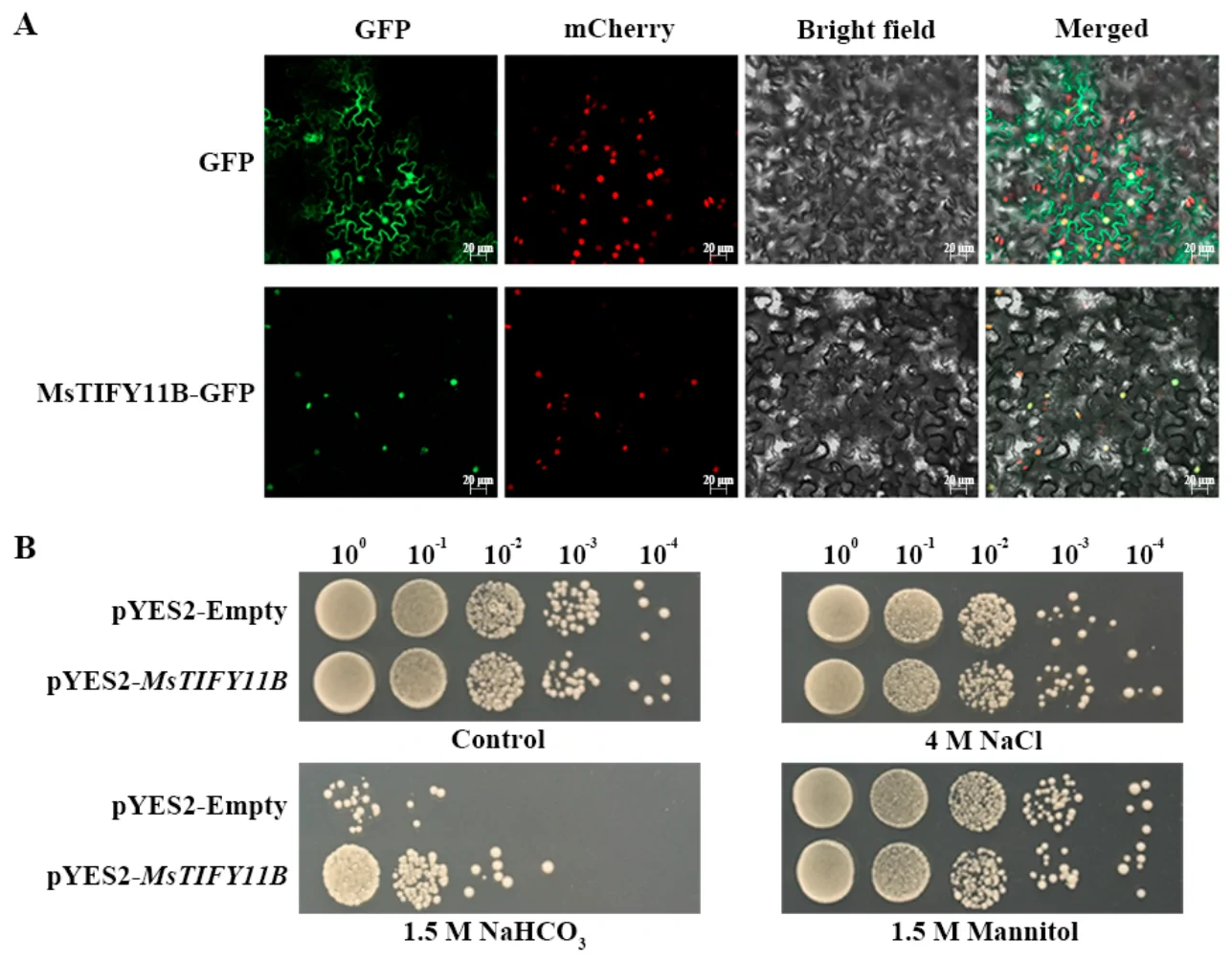
Subcellular localization of MsTIFY11B and its function in enhancing abiotic stress tolerance. (**A**) Subcellular localization of MsTIFY11B. GFP fluorescence, mCherry nuclear marker, bright-field, and merged images are labeled. The mCherry signal served as a nuclear localization marker. Scale bar = 20 μm. (**B**) Heterologous expression of *MsTIFY11B* in yeast significantly improved salt and alkaline stress tolerance. The numbers 10^0^, 10^−1^, 10^−2^, 10^−3^, and 10^−4^ indicate 10-fold serial dilutions.

To investigate the subcellular localization of MsTIFY11B, an MsTIFY11B-GFP fusion vector was constructed and transiently introduced into *Nicotiana benthamiana* leaves (Figure 7A). Results showed that the fluorescence of the 35S:MsTIFY11B-GFP fusion protein was exclusively detected in the nucleus, whereas the 35S:GFP control signal was distributed throughout the entire cell. Furthermore, to evaluate the contribution of *MsTIFY11B* to abiotic stress adaptation, we performed a preliminary functional analysis based on a yeast heterologous expression system (Figure 7B). Under normal growth conditions, yeast cells harboring the pYES2-MsTIFY11B construct exhibited a growth phenotype similar to that of the control strain harboring the empty pYES2 vector, with no significant difference. However, under high-salt (NaCl) or alkaline (NaHCO_3_) stress, the MsTIFY11B-expressing yeast cells showed better growth than the control strain at certain dilution levels, particularly at the 10^−3^ and 10^−4^ dilutions. In contrast, under mannitol-simulated drought stress, the two strains showed comparable growth performance. Overall, our findings provide preliminary evidence that *MsTIFY11B* may be involved in yeast responses to salt and alkaline stresses, whereas its role under drought stress appears to be limited.

## 3. Discussion

As a critical abiotic stress, salinity limits crop yield and degrades soil health, undermining the foundation of sustainable agricultural ecosystems [14,33]. Although transcriptomic analyses have elucidated certain adaptive mechanisms in alfalfa, the complex molecular regulatory mechanisms underlying salt-induced osmotic, ionic, and oxidative stresses remain only partially understood [29,32]. In particular, the regulatory networks governing the enhancement of alfalfa’s phytoremediation potential under saline conditions have yet to be fully explored, warranting further systematic investigation.

### 3.1. Molecular Mechanisms of Ion Homeostasis, Antioxidant Defense, and Cell Wall Remodeling in Alfalfa Under Salt Stress

When plants face salt stress, the excessive accumulation of intracellular Na^+^ and the concomitant decline in K^+^ disrupt cellular ionic homeostasis, ultimately compromising the structural integrity of the cell membrane [7,34]. Although plants employ various adaptive mechanisms to counteract this influx, they still experience a net increase in endogenous ion concentrations [35,36]. In this study, salt stress caused a significant increase in Na^+^ content alongside a marked decline in K^+^ content, subsequently disrupting the intracellular Na^+^/K^+^ balance in alfalfa (Figure 1A–C). To alleviate salt-induced oxidative stress, plants have evolved a sophisticated enzymatic antioxidant defense system, including SOD and POD [37]. Our findings revealed that during the early phase of salinity exposure (0–24 h), both SOD and POD activities increased significantly (Figure 1D,E). This rapid upregulation indicates that alfalfa can promptly activate its antioxidant machinery to scavenge ROS and alleviate cellular oxidative injury. However, as the treatment was prolonged to 72 h, both enzyme activities declined to varying degrees. This trend implies that in the later stages of stress, alfalfa might activate alternative adaptive mechanisms, thereby reducing its reliance on the aforementioned enzymatic defenses. This is consistent with previous studies reporting similar dynamic responses in plants exposed to saline conditions [38,39]. Meanwhile, GO enrichment analysis of the DEGs revealed a significant overrepresentation of oxidoreductase activity (Figure 3A–C; Table S3). This molecular fingerprint mirrors the fluctuations in antioxidant enzyme activities observed in our physiological assays, suggesting that alfalfa may activate a complex regulatory network involving redox-related genes to maintain intracellular redox homeostasis under salt stress. Furthermore, the sharp spike in MDA content during the initial phase, followed by a subsequent decline, suggests that alfalfa may exhibit regulatory responses associated with reduced lipid peroxidation levels during prolonged salt exposure (Figure 1F). However, the reduction in MDA levels at later time points does not necessarily indicate complete physiological recovery, but may reflect an adjustment of oxidative stress responses under sustained salt stress.

The plant cell wall is a highly dynamic extracellular matrix that combines exceptional plasticity with high mechanical strength [40]. Beyond its role as a physical barrier driving growth and preventing osmotic bursting, it functions as an essential, active interface for stress perception and adaptive signaling [41,42]. Upon exposure to salinity, plants actively reprogram the metabolism of major cell wall components, including lignin, pectin, cellulose, and hemicellulose, triggering a sophisticated remodeling of the extracellular framework [43,44]. These dynamic architectural adjustments underscore the essential roles of these cell wall constituents in conferring resistance to salt stress. In addition, accumulating evidence indicates that several cell wall-related genes are essential for plant adaptation to saline environments. For example, the pectin methyltransferase gene *OsTSD2* modulates ion homeostasis and salt tolerance through cell wall modification [45]. The cellulose-synthase-associated proteins CC1 and CC2 physically interact with cellulose synthase (CesA) complexes and microtubules, and loss of CC1 and CC2 function causes salt-sensitive phenotypes by disrupting microtubule dynamics and cellulose synthase complex activity [46]. The histone acetyltransferase GCN5 transcriptionally regulates cellulose synthesis genes, thereby preserving cell wall stability and enhancing plant performance under salt stress [47]. In this study, transcriptomic analysis showed that DEGs were significantly enriched in cell wall remodeling-related categories, specifically xyloglucan metabolism (GO:0010411) and cell wall biogenesis (GO:0042546) (Figure 3A–C; Table S3). This suggests that altered expression of these genes may modulate cell wall elasticity to maintain cellular structural integrity under salt stress, further highlighting the pivotal regulatory roles of cell wall-associated genes in plant salinity responses.

Carotenoids are indispensable metabolites essential for plant development, orchestrating both light-harvesting and photoprotection within the photosynthetic apparatus [48]. Beyond their structural roles, carotenoids function as essential biosynthetic precursors for stress-responsive phytohormones, including abscisic acid (ABA) and strigolactones (SLs), thereby enhancing plant resilience to adverse environments [49,50]. Accumulating evidence indicates that salt stress induces carotenoid biosynthesis [51,52], and overexpression of genes involved in carotenoid synthesis enhances salt adaptation in plants [53,54]. In our study, the carotenoid biosynthesis pathway (ko00906) exhibited sustained enrichment across all three salt-stress time points (Figure 4A–C; Table S4). Concurrently, physiological assays revealed a marked accumulation of carotenoids, an accumulation that occurred against a background of salinity-induced chlorophyll degradation (Supplementary Figure S3). These results suggest that carotenoid accumulation may be involved in the antioxidant defense and photosynthetic protection responses of alfalfa under salt stress. Mechanistically, these accumulated carotenoids may contribute to photoprotection by acting as non-enzymatic antioxidants to quench singlet oxygen and reduce potential photo-oxidative damage to photosystem II (PSII). Additionally, carotenoids may serve as metabolic precursors for ABA biosynthesis, potentially influencing downstream stress-responsive signaling pathways. Intriguingly, this metabolic shift was accompanied by a temporal transition in photosynthesis-related pathways, shifting from early carbon fixation (3 h, ko00710) to late antenna protein regulation (24 h and 72 h, ko00196) (Figure 4A–C). This programmatic cascade suggests that alfalfa dynamically remodels its light-harvesting complexes (LHCs) to dissipate excess energy and prevent photoinhibition. Collectively, our findings suggest that carotenoid metabolic adjustment, together with sequential fine-tuning of the photosynthetic machinery, may be involved in salt stress adaptation in alfalfa. Nevertheless, further functional validation is required to determine whether carotenoid accumulation plays a direct causal role in regulating salt tolerance.

### 3.2. Global Transcriptional Responses of Transcription Factors Under Salt Stress

Transcription factors serve as master molecular switches and central regulators within the intricate networks governing plant responses to abiotic stresses [55]. Accumulating evidence has demonstrated that numerous TFs belonging to the AP2/ERF, bHLH, and MYB families participate in orchestrating salt stress responses [56,57]. Here, a total of 132 salt-responsive TFs across 31 families were identified in alfalfa (Figure 5A; Table S5). Notably, the ERF family encompassed the largest group of salt-responsive members, followed by the bHLH, NAC, MYB, and HSF families. As major components of the AP2/ERF superfamily, ERFs are pivotal in modulating both plant ontogeny and stress responses. For instance, ZmEREB57 improved maize salt tolerance by modulating 12-oxo-phytodienoic acid (OPDA) synthesis via both jasmonate (JA)-dependent and -independent signaling pathways [58]. Similarly, the bHLH family serves as another critical component of salt resistance. In *Capsicum annuum*, CabHLH035 reduces salt toxicity by sustaining ion homeostasis and accelerating proline biosynthesis [59]. Furthermore, NAC TFs are indispensable for growth, development, secondary metabolism, and stress adaptation. A recent study exemplified this by demonstrating that MsNAC2a coordinates salt stress adaptation in alfalfa by controlling cellular oxidative balance and regulating hydrogen sulfide (H_2_S) metabolism [27]. Concurrently, MYB TFs are extensively involved in environmental adaptation and development, as evidenced by OsMYB2, which triggers the expression of OsANT1 to coordinately regulate development and salinity tolerance in *Oryza sativa* [60]. Although HSFs are recognized for their roles in thermotolerance, increasing attention has recently focused on their ability to alleviate salinity stress. For instance, *Arabidopsis* HSFA7b enhances salt adaptation by interacting with an E-box-like motif to activate downstream stress-responsive genes [61]. Intriguingly, a core subset of 14 TFs showed sustained differential expression across all three investigated time points, suggesting their indispensable involvement in the prolonged salinity adaptation in alfalfa (Figure 5B). Among these, five ERF genes were continuously downregulated throughout the stress duration, whereas one ERF and two HSF genes displayed sustained upregulation. This bidirectional expression profile underscores a highly sophisticated level of transcriptional fine-tuning, wherein a specific cohort of genes may act as “emergency brakes” to temporarily halt vegetative growth and conserve energy, while another cohort simultaneously activates defense-related pathways. Overall, these results demonstrate that diverse TF families orchestrate the salt stress response in alfalfa, offering detailed expression profiles and valuable candidate genes for future mechanistic validation.

### 3.3. MsTIFY11B Enhances Tolerance to Salt and Alkaline Stresses

WGCNA is a powerful approach for identifying biologically significant gene co-expression modules and screening candidate hub genes associated with target traits [62]. By evaluating intra-modular connectivity and gene-trait associations, WGCNA shifts the focus from isolated loci to coordinated networks, allowing for the precise identification of key regulatory hubs. This methodological framework has been widely applied across diverse species to uncover master regulatory genes governing complex agronomic or physiological traits [63,64,65]. To discover salt tolerance-related hub genes in alfalfa, WGCNA was performed using the FPKM values of DEGs, resulting in nine distinct co-expression modules (Figure 6A,B; Table S6). Notably, the blue module, which showed a negative correlation with Na^+^ content but a positive correlation with MDA accumulation, was highly enriched in the ribosome biogenesis pathway (Figure 6B; Table S7). Intriguingly, more than half of the nodes within this co-expression network were annotated as ribosomal proteins (Table S8), underscoring that the dynamic regulation of the translation machinery is a fundamental compensatory response to cellular stress in alfalfa. Conversely, the brown module was positively correlated with both Na^+^ accumulation and the Na^+^/K^+^ ratio (Figure 6B). This module was predominantly enriched in the plant hormone signal transduction and linolenic acid metabolism pathways (Table S7). Given that linolenic acid serves as the direct precursor for JA biosynthesis, these findings raise the possibility that salt stress may alter linolenic acid metabolism in alfalfa, potentially affecting JA biosynthesis and JA-related signaling processes that may contribute to salinity tolerance. However, the proposed link between the brown module and JA signaling remains speculative and should be regarded as a hypothesis that requires further experimental validation.

Notably, among the genes enriched in the plant hormone signal transduction pathway in the brown module, 72.7% (8 out of 11) were annotated as members of the TIFY family (Tables S7 and S8). Accumulating experimental evidence has underscored that TIFY proteins act as pivotal regulators of plant stress responses. ZmJAZ13 interacts with ZmbHLH161 and ZmA0A1D6GLB9 to improve salt adaptation in transgenic *Arabidopsis* by regulating stress-associated genes and reprogramming physiological and antioxidant defenses [66]. Conversely, *MsTIFY10a* exhibited reduced expression in alfalfa exposed to salinity, and ectopic expression of *MsTIFY10a* in tobacco markedly impaired salt tolerance in both seedlings and mature plants [67]. In this study, we identified and investigated *MsTIFY11B*, a core hub gene within the brown module. Functional validation through a yeast heterologous expression system provided preliminary evidence that MsTIFY11B may be involved in responses to salt and alkaline stresses, while its effect under drought stress appeared limited (Figure 7B). Given that yeast lacks both endogenous JA biosynthesis and the canonical JA signaling machinery, the protective effect of *MsTIFY11B* in this system cannot be explained by the classical JA pathway. Rather, this result may suggest that *MsTIFY11B* possesses intrinsic, JA-independent activities that can function in heterologous eukaryotic systems. This is consistent with a recent report that another Medicago TIFY protein, *MsTF2*, similarly enhanced stress tolerance when expressed in *Escherichia coli* and yeast, including the stress-sensitive *Δhog1* mutant expressing *MsTF2*. Such effects may involve increasing the activities of antioxidant-related enzymes and maintaining redox homeostasis [68]. Therefore, while *MsTIFY11B* acts as a transcriptional regulator in *planta*, its protective effect in yeast reflects a more fundamental biochemical property independent of JA signaling. These findings provide preliminary support for a potential role of *MsTIFY11B* in salt–alkali stress adaptation, highlighting its potential as a molecular target for improving stress resilience in alfalfa. However, a limitation of this study is that functional validation of *MsTIFY11B* was restricted to heterologous expression in yeast, which does not directly establish its biological function or regulatory importance in alfalfa. Future studies employing *MsTIFY11B* overexpression, gene silencing, or CRISPR/Cas-mediated genome editing in alfalfa, together with comprehensive physiological and molecular analyses, will be necessary to validate its function and elucidate its regulatory mechanisms under salt–alkali stress. Such investigations will further clarify the potential utility of *MsTIFY11B* as a genetic resource for improving stress tolerance in this legume species.

## 4. Materials and Methods

### 4.1. Experimental Materials and Salt Treatment

The salt-tolerant alfalfa cultivar Zhongmu No.1, which is extensively cultivated in northern China, was selected for this study [69]. Seeds were surface-sterilized and germinated in moist sand for one week. The sprouting seedlings were subsequently transferred to pots containing a 1:1 (*v*/*v*) peat–vermiculite mixture and grown in a controlled-environment greenhouse. Greenhouse conditions were maintained at 24 °C/18 °C (day/night) with a 14 h/10 h (day/night) photoperiod and 60% relative humidity. After four weeks of cultivation, the plants were transferred to plastic containers containing 50% Hoagland’s solution within a hydroponic culture system and allowed to acclimate for seven days. Salt stress was subsequently established by adding 200 mM NaCl to the 50% Hoagland’s solution, a concentration previously shown to effectively induce salt-stress responses in alfalfa [70,71,72]. Fresh leaves were sampled at 0, 3, 24, and 72 h after treatment initiation, rapidly frozen in liquid nitrogen, and stored at −80 °C. Three biological replicates were collected at each sampling time point.

### 4.2. Determination of Physiological and Biochemical Parameters

To evaluate SOD and POD activities, 0.25 g of leaves was finely pulverized using a pre-chilled mortar and liquid nitrogen. The powder was transferred into 4 mL of 50 mM phosphate buffer (pH 7.8) in a 10 mL centrifuge tube, thoroughly mixed by vortexing, and centrifuged at 13,000× *g* for 20 min at 4 °C. The collected supernatant was subsequently subjected to SOD and POD activity assays following an established protocol [73].

To quantify MDA content, 0.2 g of leaves was finely homogenized and extracted with 150 mM phosphate buffer (pH 7.0). Following centrifugation of the homogenate at 13,000× *g* for 20 min, the supernatant was mixed with a solution containing 0.5% 2-thiobarbituric acid and 20% trichloroacetic acid. The reaction solution was incubated at 95 °C for 30 min, cooled to room temperature, and subjected to a second centrifugation at 4000× *g* for 20 min. MDA content was quantified as described previously [74].

For the quantification of Na^+^ and K^+^, fresh alfalfa leaves were dried at 105 °C for 30 min and then placed at 75 °C for 2 days. Subsequently, the dried sample (0.1 g) was finely pulverized, mixed with 5 mL of 95% sulfuric acid and 1 mL of 30% hydrogen peroxide, and digested at 420 °C for 2.5 h in a graphite digestion system (SH220N, Jinan, China). After digestion, the resulting digestion solution was diluted 5- to 10-fold, and Na^+^ and K^+^ contents were quantified using a flame spectrophotometer (F-500, Shanghai, China) according to an established protocol [75].

To quantify photosynthetic pigments, fresh leaves were finely pulverized using liquid nitrogen and extracted with 30 mL of 95% ethanol in the dark for 2 days. The absorbance values of the extracts were determined at 665 nm, 649 nm, and 470 nm using a spectrophotometer, and chlorophyll and carotenoid contents were calculated using a previously established method [76,77].

### 4.3. RNA-Seq Library Preparation and Sequencing

To capture the temporal dynamics of the alfalfa transcriptomic response from early perception and signaling to subsequent metabolic adjustment and long-term adaptation, leaf samples from salt-stressed Zhongmu No.1 plants were harvested at 0, 3, 24, and 72 h after treatment (three biological replicates per time point). Total RNA was extracted using TRIzol reagent. After assessment of RNA quality, including purity, concentration, and RNA integrity, transcriptome libraries were prepared using the VAHTS Universal V6 RNA-seq Library Prep Kit. Subsequently, the prepared libraries were sequenced on the Illumina NovaSeq 6000 platform (OEbiotech, Shanghai, China), generating 150 bp paired-end reads.

### 4.4. Transcriptome Analysis

Raw reads were processed using fastp software (v0.20.1) to remove adapter sequences and filter out low-quality reads, generating high-quality clean reads for subsequent analyses [78]. The clean reads were subsequently aligned to the alfalfa reference genome (https://figshare.com/articles/dataset/Medicago_sativa_genome_and_annotation_files/12623960, accessed on 2 August 2026) using HISAT2 (v2.1.0) [79]. The resulting SAM files were converted to BAM format and sorted using SAMtools (v1.9) [80]. Transcript assembly and abundance estimation were performed using StringTie (v2.2.1) with default parameters, and gene expression levels were quantified as Fragments Per Kilobase of exon model per Million mapped reads (FPKM) [81]. For differential expression analysis, read counts were extracted using the prepDE.py script provided with StringTie (v2.2.1) [81]. DEGs were identified using the DESeq2 package (v1.22.2) based on the count matrix, with |log2FoldChange| ≥ 1 and adjusted *p*-value < 0.05 as the thresholds [82]. The identified DEGs were subsequently subjected to Gene Ontology (GO) and Kyoto Encyclopedia of Genes and Genomes (KEGG) enrichment analyses [83,84]. Data visualization was performed using R (v4.2.2).

### 4.5. Transcription Factor Identification and Analysis

To identify salt stress-induced transcription factors, we performed BLASTP (v2.2.27) searches against the PlantTFDB database using the differentially expressed alfalfa protein sequences as queries [85]. The TFs identified at different time points were then statistically analyzed. Their expression levels were extracted from the transcriptomic dataset using a Perl script and visualized using the pheatmap (v1.0.12) package.

### 4.6. WGCNA of Salt-Responsive Genes in Alfalfa

The FPKM expression profiles of all 2625 non-redundant DEGs across the 12 samples were extracted for co-expression network construction using WGCNA [62]. Network construction was performed using a soft-thresholding power of 7, followed by the calculation of an unsigned topological overlap matrix (TOM). The minimum number of genes in each module was set to 30, with a cut height of 0.3 for module merging. Module eigengenes were subsequently calculated to assess module–trait relationships across the 12 samples. To characterize the functions of each module, KEGG pathway analysis was performed using each module’s gene sets. Subsequently, the blue module (edges with weights > 0.65) and the brown module (edges with weights > 0.50) were analyzed and visualized using the Network Analyzer tool and Cytoscape 3.8.0 [86]. Candidate hub genes within the key modules were identified based on their intramodular connectivity, as reflected by the degree values calculated from the co-expression network.

### 4.7. RT-qPCR Analysis

To assess the reliability of the RNA-Seq results, nine salt-responsive genes were randomly selected for RT-qPCR validation. Primers were designed using the Design Primer tool available in the MODMS database [87]. cDNA was synthesized using a first-strand cDNA synthesis kit (Takara Bio Inc., Kusatsu, Shia, Japan). RT-qPCR assays were conducted using a CFX96 Touch™ Real-Time PCR Detection System (Bio-Rad Laboratories, Inc., Hercules, CA, USA). The relative transcript abundance of these nine genes was calculated using the 2^−∆∆Ct^ method [88], with alfalfa *ACT2* as the housekeeping gene [30]. The detailed primer information is provided in Table S7. Data visualization was performed using Origin 8.6 software.

### 4.8. Subcellular Localization and Functional Analysis of MsTIFY11B

To investigate the subcellular localization of MsTIFY11B, primers were designed using CE Design V1.04 software and used for PCR amplification (Table S9). The CDS fragment of *MsTIFY11B* lacking the stop codon was amplified and ligated into the 35S:GFP expression vector. The resulting plasmid was transformed into *E. coli* DH5α, and positive clones were verified by sequencing. The verified plasmid was introduced into *Agrobacterium tumefaciens* strain GV3101 and infiltrated into *N. benthamiana* leaves via agroinfiltration for transient expression. A nuclear-localized marker protein was co-expressed as a nuclear localization reference. After 48–72 h of incubation, subcellular fluorescence patterns were observed using a Zeiss LSM710 laser scanning confocal microscope (Oberkochen, Germany).

To investigate the potential function of *MsTIFY11B*, the full-length CDS was cloned into the pYES2 vector. The resulting plasmid and the empty vector were transformed into *Saccharomyces cerevisiae* strain INVSc1. Yeast transformants were screened on SD-Ura solid medium supplemented with 2% (*w*/*v*) glucose at 30 °C for 1 day. Subsequently, positive clones were cultured overnight in SD-Ura liquid medium containing 2% (*w*/*v*) galactose at 30 °C to induce protein expression. When the cultures reached an OD_600_ of 2.0, serial 10-fold dilutions were prepared, and aliquots of each dilution were plated on SD-Ura medium containing 0 or 4 M NaCl, 0 or 1.5 M NaHCO_3_, or 0 or 1.5 M mannitol. After incubation at 30 °C for 3–4 days, the plates were photographed.

### 4.9. Statistical Analysis

All statistical analyses were conducted using SPSS Statistics 26.0 (IBM Corp., Armonk, NY, USA). Treatment differences were evaluated using one-way analysis of variance (ANOVA), with multiple comparisons performed using Fisher’s least significant difference (LSD) post hoc test.

## 5. Conclusions

This study successfully integrated physiological profiling, time-course RNA-seq, and WGCNA to elucidate the regulatory network governing salt stress responses in alfalfa. Our results demonstrate that alfalfa orchestrates physiological adaptations, carotenoid accumulation, and temporally distinct transcriptional programs to counteract salt-induced osmotic and oxidative damages. Notably, co-expression network analysis identified and characterized a nuclear-localized hub gene, *MsTIFY11B*, which is engaged in plant hormone signal transduction. Heterologous expression in yeast confirmed that *MsTIFY11B* functions as a potent and specific positive regulator of salt and alkali tolerance, while offering only limited protection against drought stress. Collectively, these results broaden our understanding of the functional diversity of TIFY family members in alfalfa stress responses and identify MsTIFY11B as a promising candidate for further investigation of its potential role in improving alfalfa performance under salt–alkali stress and marginal soil conditions.

## Data Availability

The RNA-seq raw data generated in this study have been deposited in the National Genomics Data Center (NGDC) under BioProject accession number PRJCA070763. All analyzed data are included in this article and its Supplementary Information Files.

## Supplementary Materials

The following supporting information can be downloaded at: https://www.mdpi.com/article/10.3390/plants15172714/s1, Figure S1. Transcriptome sequencing quality and correlation analyses of samples under salt stress. (A) PCA analysis of the transcriptomic data. (B) Pearson correlation coefficient analysis of twelve biological samples. Figure S2. Expression profiles of nine genes and the correlation between RNA-Seq data and RT-qPCR data. (A) MsG0880042776.01. (B) MsG0180003410.01. (C) MsG0680033884.01. (D) MsG0380017116.01. (E) MsG0280009781.01. (F) MsG0880045939.01. (G) MsG0580024357.01. (H) MsG0580029673.01. (I) MsG0280009931.01. The RNA-Seq (fold change) and RT-qPCR (relative expression) data of nine genes were plotted using Origin. (J) The correlation between RNA-Seq data and RT-qPCR validation of nine genes. Figure S3. Chlorophyll and carotenoid contents at different time points under salt stress. (A) Chlorophyll content. (B) Carotenoid content. Data are presented as means ± SD of three independent biological replicates. Statistical significance was determined by one-way ANOVA followed by Fisher’s LSD post hoc test. Different lowercase letters indicate statistically significant differences between groups (*p* < 0.05). Figure S4: Phylogenetic analysis of MsTIFY11B and 18 Arabidopsis TIFY family members. Multiple sequence alignment was performed on 19 TIFY proteins, and a phylogenetic tree was constructed using MEGA7 with the neighbor-joining method and 1000 bootstrap replicates. Table S1. Summary of transcriptome data quality. Table S2. Differentially expressed genes in alfalfa leaves under NaCl treatment for 3, 24, and 72 h. Table S3. GO analysis of salt-responsive DEGs. Table S4. KEGG pathway analysis of salt-responsive DEGs. Table S5. Differentially expressed transcription factors under salt stress. Table S6. WGCNA analysis of 2625 non-redundant differentially expressed genes and their corresponding modules. Table S7. KEGG pathway enrichment analysis of blue and brown module genes. Table S8. Gene expression levels and functional annotations for the blue and brown co-expression networks. Table S9. Primers used in this study.

## References

[1] You X., Fan N., Zhang Y., Sun L., Su L., Wen W., Lv A., Dai X., Gao L., Shi F. (2025). The MsNAC73-MsMPK3 Complex Modulates Salt Tolerance and Shoot Branching of Alfalfa via Activating *MsPG2* and *MsPAE12* Expressions. Plant Biotechnol. J..

[2] Shi Y., Chen R., Jiang K., Jiang A., Yang J., Cui H., Chen M. (2026). Key metabolites secreted by *Chlorella vulgaris* alleviate salt stress in soybean seedlings. J. Integr. Plant Biol..

[3] Abiala M.A., Abdelrahman M., Burritt D.J., Tran L.-S.P. (2018). Salt stress tolerance mechanisms and potential applications of legumes for sustainable reclamation of salt-degraded soils. Land Degrad. Dev..

[4] Cooper M., Messina C.D. (2023). Breeding crops for drought-affected environments and improved climate resilience. Plant Cell.

[5] Ma L., Li X., Zhang J., Yi D., Li F., Wen H., Liu W., Wang X. (2023). *MsWRKY33* increases alfalfa (*Medicago sativa* L.) salt stress tolerance through altering the ROS scavenger via activating *MsERF5* transcription. Plant Cell Environ..

[6] Yang Y., Guo Y. (2018). Unraveling salt stress signaling in plants. J. Integr. Plant Biol..

[7] Basu S., Kumar A., Benazir I., Kumar G. (2021). Reassessing the role of ion homeostasis for improving salinity tolerance in crop plants. Physiol. Plant..

[8] Wang P., Liu W.C., Han C., Wang S., Bai M.Y., Song C.P. (2024). Reactive oxygen species: Multidimensional regulators of plant adaptation to abiotic stress and development. J. Integr. Plant Biol..

[9] Miller G., Suzuki N., Ciftci-Yilmaz S., Mittler R. (2010). Reactive oxygen species homeostasis and signalling during drought and salinity stresses. Plant Cell Environ..

[10] Zhao C., Zhang H., Song C., Zhu J.K., Shabala S. (2020). Mechanisms of Plant Responses and Adaptation to Soil Salinity. Innovation.

[11] Song Y., Lv J., Ma Z., Dong W. (2019). The mechanism of alfalfa (*Medicago sativa* L.) response to abiotic stress. Plant Growth Regul..

[12] van Zelm E., Zhang Y., Testerink C. (2020). Salt Tolerance Mechanisms of Plants. Annu. Rev. Plant Biol..

[13] Zhu J.K. (2016). Abiotic Stress Signaling and Responses in Plants. Cell.

[14] Ma L., Li J., Li J., Huo Y., Yang Y., Jiang C., Guo Y. (2026). Plant salt-tolerance mechanisms: Classic signaling pathways, emerging frontiers, and future perspectives. Mol. Plant.

[15] Huang G., Wang X., Liu C., He K., Hou X.L., Luo H., Zhang S., You C., Jia Y., Wang F. (2025). Genomic Variation Underpins Genetic Divergence and Differing Salt Resilience in *Sesbania bispinosa*. Adv. Sci..

[16] Estrada Y., Fernández-Ojeda A., Morales B., Egea-Fernández J.M., Flores F.B., Bolarín M.C., Egea I. (2021). Unraveling the Strategies Used by the Underexploited Amaranth Species to Confront Salt Stress: Similarities and Differences with Quinoa Species. Front. Plant Sci..

[17] Iqbal M.S., Clode P.L., Malik A.I., Erskine W., Kotula L. (2024). Salt tolerance in mungbean is associated with controlling Na and Cl transport across roots, regulating Na and Cl accumulation in chloroplasts and maintaining high K in root and leaf mesophyll cells. Plant Cell Environ..

[18] Xu Q., Gan J., Zhou Z., Zhou T., Lu R., Liu N., Hu L. (2025). Dynamic transcriptomics and physiological insights reveal multi-tissue salt adaptation mechanisms in *Amaranthus hypochondriacus* across stress gradients. Plant Cell Rep..

[19] Hu Y., Wang D., Zhang X., Lv X., Li B. (2025). Current progress in deciphering the molecular mechanisms underlying plant salt tolerance. Curr. Opin. Plant Biol..

[20] Zhang F., Ji B., Wu S., Zhang J., Zhang H., Wang F., Song B., Sang Q., Huang W., Yan S. (2025). High-resolution time-series transcriptomic and metabolomic profiling reveals the regulatory mechanism underlying salt tolerance in maize. Genome Biol..

[21] Vilarrasa-Blasi J., Vellosillo T., Jinkerson R.E., Fauser F., Xiang T., Minkoff B.B., Wang L., Kniazev K., Guzman M., Osaki J. (2024). Multi-omics analysis of green lineage osmotic stress pathways unveils crucial roles of different cellular compartments. Nat. Commun..

[22] Zhou Y., Wang D., Wang H., Qiao Y., Zhao P., Cao Y., Liu X., Yang Y., Lin X., Xu S. (2025). Integrative omics of the genetic basis for wheat WUE and drought resilience reveal the function of TaMYB7-A1. Nat. Commun..

[23] Zhu Z., Zhou Y., Liu X., Meng F., Xu C., Chen M. (2025). Integrated transcriptomic and metabolomic analyses uncover the key pathways of *Limonium bicolor* in response to salt stress. Plant Biotechnol. J..

[24] Li S., Li Y., Qiang W., Zhang Y., Yuan M., Wei S., Xu C., Chen Q., Hu Z., Zhao Y. (2026). Transcriptome analysis uncovers salt stress-responsive mechanisms and *GmCML48* as a key candidate gene in soybean. Plant Stress.

[25] Wang T., Yang J., Cao J., Zhang Q., Liu H., Li P., Huang Y., Qian W., Bi X., Wang H. (2025). MsbZIP55 regulates salinity tolerance by modulating melatonin biosynthesis in alfalfa. Plant Biotechnol. J..

[26] Guo W., Chen J., Liu L., Ren Y., Guo R., Ding Y., Li Y., Chai J., Sun Y., Guo C. (2024). *MsMIOX2*, encoding a *MsbZIP53*-activated myo-inositol oxygenase, enhances saline-alkali stress tolerance by regulating cell wall pectin and hemicellulose biosynthesis in alfalfa. Plant J..

[27] Lu S., Sun Y., Wang Z., Yu W., Dong Z., Li S., Ma L., Su K., Zhang H., Wang Z.Y. (2025). Alfalfa Transcription Factor MsNAC2a Orchestrates the Homeostasis of Salt Stress Responses via Reactive Oxygen Species Accumulation and Hydrogen Sulphide Depletion. Plant Biotechnol. J..

[28] Lin S., Yang J., Liu Y., Zhang W. (2024). *MsSPL12* is a positive regulator in alfalfa (*Medicago sativa* L.) salt tolerance. Plant Cell Rep..

[29] Liu L., Si L., Zhang L., Guo R., Wang R., Dong H., Guo C. (2024). Metabolomics and transcriptomics analysis revealed the response mechanism of alfalfa to combined cold and saline-alkali stress. Plant J..

[30] Song Y., Tang H., Zhang Z., Sun X., Ding X., Guo X., Wang Q., Chen J., Dong W. (2025). A Novel MsEOBI-*MsPAL1* Module Enhances Salinity Stress Tolerance, Floral Scent Emission and Seed Yield in Alfalfa. Plant Cell Environ..

[31] Su L., Lv A., Wen W., Fan N., You X., Gao L., Zhou P., Shi F., An Y. (2025). MsMYB206-MsMYB450-MsHY5 complex regulates alfalfa tolerance to salt stress via regulating flavonoid biosynthesis during the day and night cycles. Plant J..

[32] Guo R., Liu L., Li J., Qu H., Guo W., Zhang L., Yang D., Wang R., Guo C. (2026). Metabolo-Transcriptomics Analyses Reveal Alfalfa Adaptation to Combined Saline-Alkali and Low-Temperature Stress in the Field. Plant Biotechnol. J..

[33] Yuan H., Cheng M., Wang R., Wang Z., Fan F., Wang W., Si F., Gao F., Li S. (2024). miR396b/*GRF6* module contributes to salt tolerance in rice. Plant Biotechnol. J..

[34] Sun J., Agneessens J., Topping J.F., Lindsey K. (2026). The vesicle trafficking R-SNARE VAMP714 connects auxin responses, ROS and ion homeostasis in Arabidopsis under salt stress. New Phytol..

[35] Ramakrishna P., Gámez-Arjona F.M., Bellani E., Martin-Olmos C., Escrig S., De Bellis D., De Luca A., Pardo J.M., Quintero F.J., Genoud C. (2025). Elemental cryo-imaging reveals SOS1-dependent vacuolar sodium accumulation. Nature.

[36] Liu H.S., Liu Q., Hepworth S.R., Li P.Q., Huang J., Zhang R.X., Ma C.M., Gao T.G., Ma H.P., Ke J. (2025). ZxNHX1 from a xerophyte outperforms AtNHX1 in sequestering Na(+) into vacuoles to enhance plant stress resistance and yield. Plant Biotechnol. J..

[37] Hasanuzzaman M., Bhuyan M., Zulfiqar F., Raza A., Mohsin S.M., Mahmud J.A., Fujita M., Fotopoulos V. (2020). Reactive Oxygen Species and Antioxidant Defense in Plants under Abiotic Stress: Revisiting the Crucial Role of a Universal Defense Regulator. Antioxidants.

[38] Ji X., Tang J., Zhang J. (2022). Effects of Salt Stress on the Morphology, Growth and Physiological Parameters of *Juglans microcarpa* L. Seedlings. Plants.

[39] Guo X., Ahmad N., Zhao S., Zhao C., Zhong W., Wang X., Li G. (2022). Effect of Salt Stress on Growth and Physiological Properties of Asparagus Seedlings. Plants.

[40] Wang G.L., Ren X.Q., Liu J.X., Yang F., Wang Y.P., Xiong A.S. (2019). Transcript profiling reveals an important role of cell wall remodeling and hormone signaling under salt stress in garlic. Plant Physiol. Biochem..

[41] Oliveira D.M., Mota T.R., Salatta F.V., Sinzker R.C., Končitíková R., Kopečný D., Simister R., Silva M., Goeminne G., Morreel K. (2020). Cell wall remodeling under salt stress: Insights into changes in polysaccharides, feruloylation, lignification, and phenolic metabolism in maize. Plant Cell Environ..

[42] Lou T., Lv S., Wang J., Wang D., Lin K., Zhang X., Zhang B., Guo Z., Yi Z., Li Y. (2024). Cell size and xylem differentiation regulating genes from *Salicornia europaea* contribute to plant salt tolerance. Plant Cell Environ..

[43] van der Cruijsen K., Al Hassan M., van Erven G., Kollerie N., van Lent B., Dechesne A., Dolstra O., Paulo M.J., Trindade L.M. (2024). Salt stress alters the cell wall components and structure in *Miscanthus sinensis* stems. Physiol. Plant..

[44] Dabravolski S.A., Isayenkov S.V. (2023). The regulation of plant cell wall organisation under salt stress. Front. Plant Sci..

[45] Fang C., Li K., Wu Y., Wang D., Zhou J., Liu X., Li Y., Jin C., Liu X., Mur L.A.J. (2019). *OsTSD2*-mediated cell wall modification affects ion homeostasis and salt tolerance. Plant Cell Environ..

[46] Endler A., Kesten C., Schneider R., Zhang Y., Ivakov A., Froehlich A., Funke N., Persson S. (2015). A Mechanism for Sustained Cellulose Synthesis during Salt Stress. Cell.

[47] Zheng M., Liu X., Lin J., Liu X., Wang Z., Xin M., Yao Y., Peng H., Zhou D.X., Ni Z. (2019). Histone acetyltransferase GCN5 contributes to cell wall integrity and salt stress tolerance by altering the expression of cellulose synthesis genes. Plant J..

[48] Sun T., Tadmor Y., Li L. (2020). Pathways for Carotenoid Biosynthesis, Degradation, and Storage. Methods Mol. Biol..

[49] Ezquerro M., Li C., Pérez-Pérez J., Burbano-Erazo E., Barja M.V., Wang Y., Dong L., Lisón P., López-Gresa M.P., Bouwmeester H.J. (2023). Tomato geranylgeranyl diphosphate synthase isoform 1 is involved in the stress-triggered production of diterpenes in leaves and strigolactones in roots. New Phytol..

[50] Felemban A., Moreno J.C., Mi J., Ali S., Sham A., AbuQamar S.F., Al-Babili S. (2024). The apocarotenoid β-ionone regulates the transcriptome of *Arabidopsis thaliana* and increases its resistance against *Botrytis cinerea*. Plant J..

[51] Zhao Y.-H., Deng Y.-J., Wang Y.-H., Lou Y.-R., He L.-F., Liu H., Li T., Yan Z.-M., Zhuang J., Xiong A.-S. (2022). Changes in Carotenoid Concentration and Expression of Carotenoid Biosynthesis Genes in *Daucus carota* Taproots in Response to Increased Salinity. Horticulturae.

[52] Leiva-Ampuero A., Agurto M., Matus J.T., Hoppe G., Huidobro C., Inostroza-Blancheteau C., Reyes-Díaz M., Stange C., Canessa P., Vega A. (2020). Salinity impairs photosynthetic capacity and enhances carotenoid-related gene expression and biosynthesis in tomato (*Solanum lycopersicum* L. cv. Micro-Tom). PeerJ.

[53] Li C., Wang C., Cheng Z., Li Y., Li W. (2024). Carotenoid biosynthesis genes *LcLCYB*, *LcLCYE*, and *LcBCH* from wolfberry confer increased carotenoid content and improved salt tolerance in tobacco. Sci. Rep..

[54] Li R., Kang C., Song X., Yu L., Liu D., He S., Zhai H., Liu Q. (2017). A ζ-carotene desaturase gene, *IbZDS*, increases β-carotene and lutein contents and enhances salt tolerance in transgenic sweetpotato. Plant Sci..

[55] Wei H., Wang X., Wang K., Tang X., Zhang N., Si H. (2024). Transcription factors as molecular switches regulating plant responses to drought stress. Physiol. Plant..

[56] Admas T., Shu J., Shalmani A., Pan R., Zhang W. (2025). Salt stress-responsive transcription factors provide insights to enhance barley improvement: A review. Planta.

[57] Aizaz M., Lubna, Jan R., Asaf S., Bilal S., Kim K.M., Al-Harrasi A. (2024). Regulatory Dynamics of Plant Hormones and Transcription Factors under Salt Stress. Biology.

[58] Zhu J., Wei X., Yin C., Zhou H., Yan J., He W., Yan J., Li H. (2023). ZmEREB57 regulates OPDA synthesis and enhances salt stress tolerance through two distinct signalling pathways in *Zea mays*. Plant Cell Environ..

[59] Zhang H., Guo J., Chen X., Zhou Y., Pei Y., Chen L., Ul Haq S., Lu M., Gong H., Chen R. (2022). Pepper bHLH transcription factor *CabHLH035* contributes to salt tolerance by modulating ion homeostasis and proline biosynthesis. Hortic. Res..

[60] Nie S., Huang W., He C., Wu B., Duan H., Ruan J., Zhao Q., Fang Z. (2025). Transcription factor OsMYB2 triggers amino acid transporter *OsANT1* expression to regulate rice growth and salt tolerance. Plant Physiol..

[61] Zang D., Wang J., Zhang X., Liu Z., Wang Y. (2019). Arabidopsis heat shock transcription factor HSFA7b positively mediates salt stress tolerance by binding to an E-box-like motif to regulate gene expression. J. Exp. Bot..

[62] Langfelder P., Horvath S. (2008). WGCNA: An R package for weighted correlation network analysis. BMC Bioinform..

[63] Mutinda S., Mobegi F.M., Hale B., Dayou O., Ateka E., Wijeratne A., Wicke S., Bellis E.S., Runo S. (2023). Resolving intergenotypic *Striga* resistance in sorghum. J. Exp. Bot..

[64] Ma L., Zhang M., Chen J., Qing C., He S., Zou C., Yuan G., Yang C., Peng H., Pan G. (2021). GWAS and WGCNA uncover hub genes controlling salt tolerance in maize (*Zea mays* L.) seedlings. Theor. Appl. Genet..

[65] Qi Z., Zhang Z., Wang Z., Yu J., Qin H., Mao X., Jiang H., Xin D., Yin Z., Zhu R. (2018). Meta-analysis and transcriptome profiling reveal hub genes for soybean seed storage composition during seed development. Plant Cell Environ..

[66] Zhang S., Zheng D., Gao Y., She M., Wu Z., Lu Y., Zhang Z. (2025). The TIFY transcription factor ZmJAZ13 enhances plant tolerance to drought and salt stress by interacting with ZmbHLH161 and ZmA0A1D6GLB9. Plant Sci..

[67] Chen Q., Zhang Y., Tian Y., Xu J., Fu Q.W., Li Z.Y., Shi F.L., Gao C.P., Zhang Z.Q. (2026). *MsTIFY10a* gene from alfalfa negatively regulates drought and salt tolerance in transgenic tobacco. J. Plant Physiol..

[68] Wang X., Lan Q., Li X., Zhao Y. (2026). Genomic analysis of *TIFY* genes in two *Medicago* and insight into MsTF2-mediated abiotic stress tolerance. Plant Cell Rep..

[69] Lei Y., Xu Y., Hettenhausen C., Lu C., Shen G., Zhang C., Li J., Song J., Lin H., Wu J. (2018). Comparative analysis of alfalfa (*Medicago sativa* L.) leaf transcriptomes reveals genotype-specific salt tolerance mechanisms. BMC Plant Biol..

[70] Xiong X., Wei Y., Chen J., Liu N., Zhang Y. (2020). Transcriptome analysis of genes and pathways associated with salt tolerance in alfalfa under non-uniform salt stress. Plant Physiol. Biochem..

[71] Chen L., Zhang Y., Ma X., Bi J., He F., Zhao L., Lei H., Xiao Z., Wang X., Zhang T. (2026). Conditionally Significant eQTL and TWAS Analyses Identify MsRD26 as a Major Candidate Positive Regulator of Salt Tolerance in *Medicago sativa* L.. Adv. Sci..

[72] Wang Y., Wu W., Liang F., Zhao T., Li S., Yang M., Leng F., Zhu X., Wang X. (2026). Integrated multi-omics reveals divergent salt stress response mechanisms in three alfalfa (*Medicago sativa* L.) cultivars. Plant Sci..

[73] Shao A., Wang H., Xu X., Li X., Amombo E., Fu J. (2022). Moderately Reducing Nitrogen Application Ameliorates Salt-Induced Growth and Physiological Damage on Forage Bermudagrass. Front. Plant Sci..

[74] Yin Y., Fan S., Li S., Amombo E., Fu J. (2023). Involvement of cell cycle and ion transferring in the salt stress responses of alfalfa varieties at different development stages. BMC Plant Biol..

[75] Cui X., Chen J., Li S., Shao A., Fu J. (2025). Growth and physiological characteristics of forage bermudagrass in response to salt stress. BMC Plant Biol..

[76] Fargasová A., Molnárová M. (2010). Assessment of Cr and Ni phytotoxicity from cutlery-washing waste-waters using biomass and chlorophyll production tests on mustard *Sinapis alba* L. seedlings. Environ. Sci. Pollut. Res. Int..

[77] Lichtenthaler H.K. (1987). Chlorophylls and carotenoids: Pigments of photosynthetic biomembranes. Methods Enzymol..

[78] Chen S., Zhou Y., Chen Y., Gu J. (2018). fastp: An ultra-fast all-in-one FASTQ preprocessor. Bioinformatics.

[79] Shen C., Du H., Chen Z., Lu H., Zhu F., Chen H., Meng X., Liu Q., Liu P., Zheng L. (2020). The Chromosome-Level Genome Sequence of the Autotetraploid Alfalfa and Resequencing of Core Germplasms Provide Genomic Resources for Alfalfa Research. Mol. Plant.

[80] Pertea M., Pertea G.M., Antonescu C.M., Chang T.C., Mendell J.T., Salzberg S.L. (2015). StringTie enables improved reconstruction of a transcriptome from RNA-seq reads. Nat. Biotechnol..

[81] Li H., Handsaker B., Wysoker A., Fennell T., Ruan J., Homer N., Marth G., Abecasis G., Durbin R. (2009). The Sequence Alignment/Map format and SAMtools. Bioinformatics.

[82] Love M.I., Huber W., Anders S. (2014). Moderated estimation of fold change and dispersion for RNA-seq data with DESeq2. Genome Biol..

[83] Ashburner M., Ball C.A., Blake J.A., Botstein D., Butler H., Cherry J.M., Davis A.P., Dolinski K., Dwight S.S., Eppig J.T. (2000). Gene ontology: Tool for the unification of biology. The Gene Ontology Consortium. Nat. Genet..

[84] Kanehisa M., Furumichi M., Sato Y., Ishiguro-Watanabe M., Tanabe M. (2021). KEGG: Integrating viruses and cellular organisms. Nucleic Acids Res..

[85] Jin J., Tian F., Yang D.-C., Meng Y.-Q., Kong L., Luo J., Gao G. (2016). PlantTFDB 4.0: Toward a central hub for transcription factors and regulatory interactions in plants. Nucleic Acids Res..

[86] Shannon P., Markiel A., Ozier O., Baliga N.S., Wang J.T., Ramage D., Amin N., Schwikowski B., Ideker T. (2003). Cytoscape: A software environment for integrated models of biomolecular interaction networks. Genome Res..

[87] Fang L., Liu T., Li M., Dong X., Han Y., Xu C., Li S., Zhang J., He X., Zhou Q. (2023). MODMS: A multi-omics database for facilitating biological studies on alfalfa (*Medicago sativa* L.). Hortic. Res..

[88] Livak K.J., Schmittgen T.D. (2001). Analysis of relative gene expression data using real-time quantitative PCR and the 2(-Delta Delta C(T)) Method. Methods.

