# Supplementary material for: Time-Course Transcriptomic Analysis Identifies MsTIFY11B as a Promising Candidate Regulator of Salt–Alkali Tolerance in Alfalfa (Medicago sativa L.)
Source: Plants (Basel). 2026 Sep 4;15(17):2714. doi: 10.3390/plants15172714 (PMC13567717; doi:10.3390/plants15172714)
Supplement: Supplementary file 1 [file plants-15-02714-s001.zip › Supplemental Figures.pdf]

Title:

Time-course transcriptomic analysis identifies *MsTIFY11B* as a promising candidate regulator of salt–alkali tolerance in alfalfa (*Medicago sativa* L.)

Authors:

Linfei Cheng [1 †], Yanfeng Liu [2 †], Qingchun Liu [1], Dong Li [2], Yanan Chen [2], Jiadong Chen [2], Yang He [1], Wei Wang [1 *]

Supporting information:

Figure S1. Transcriptome sequencing quality and correlation analyses of samples under salt stress. (A) PCA analysis of the transcriptomic data. (B) Pearson correlation coefficient analysis of twelve biological samples.

Figure S2. Expression profiles of nine genes and the correlation between RNA-Seq data and RT-qPCR data. (A) MsG0880042776.01. (B) MsG0180003410.01. (C) MsG0680033884.01. (D) MsG0380017116.01. (E) MsG0280009781.01. (F) MsG0880045939.01. (G) MsG0580024357.01. (H) MsG0580029673.01. (I) MsG0280009931.01. The RNA-Seq (fold change) and RT-qPCR (relative expression) data of nine genes were plotted using Origin. (J) The correlation between RNA-Seq data and RT-qPCR validation of nine genes.

Figure S3. Chlorophyll and carotenoid contents at different time points under salt stress. (A) Chlorophyll content. (B) Carotenoid content. Data are presented as means ± SD of three independent biological replicates. Statistical significance was determined by one-way ANOVA followed by Fisher's LSD post hoc test. Different lowercase letters indicate statistically significant differences between groups ($p < 0.05$).

Figure S4. Phylogenetic analysis of MsTIFY11B and 18 Arabidopsis TIFY family members. Multiple sequence alignment was performed on 19 TIFY proteins, and a phylogenetic tree was constructed using MEGA7 with the neighbor-joining method and 1000 bootstrap replicates.

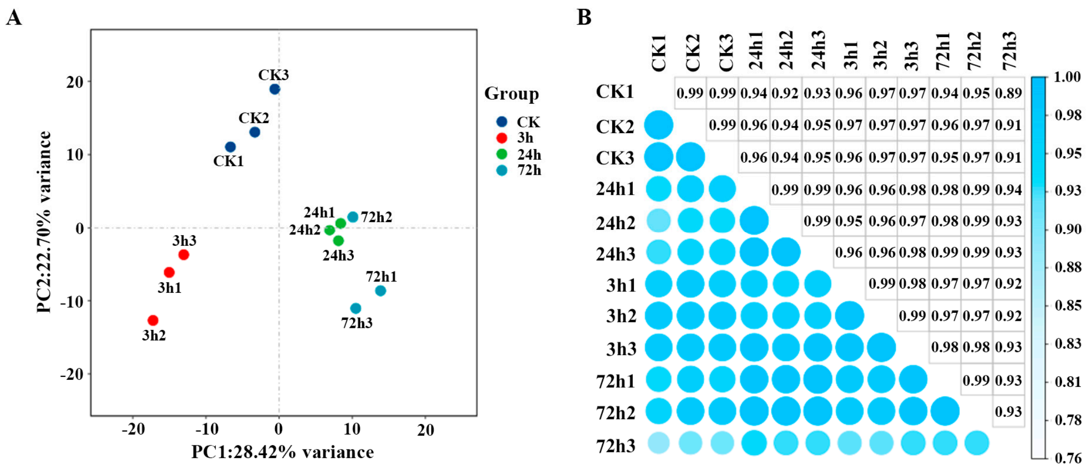

Figure S1. Transcriptome sequencing quality and correlation analyses of samples under salt stress. (A) PCA analysis of the transcriptomic data. (B) Pearson correlation coefficient analysis of twelve biological samples.

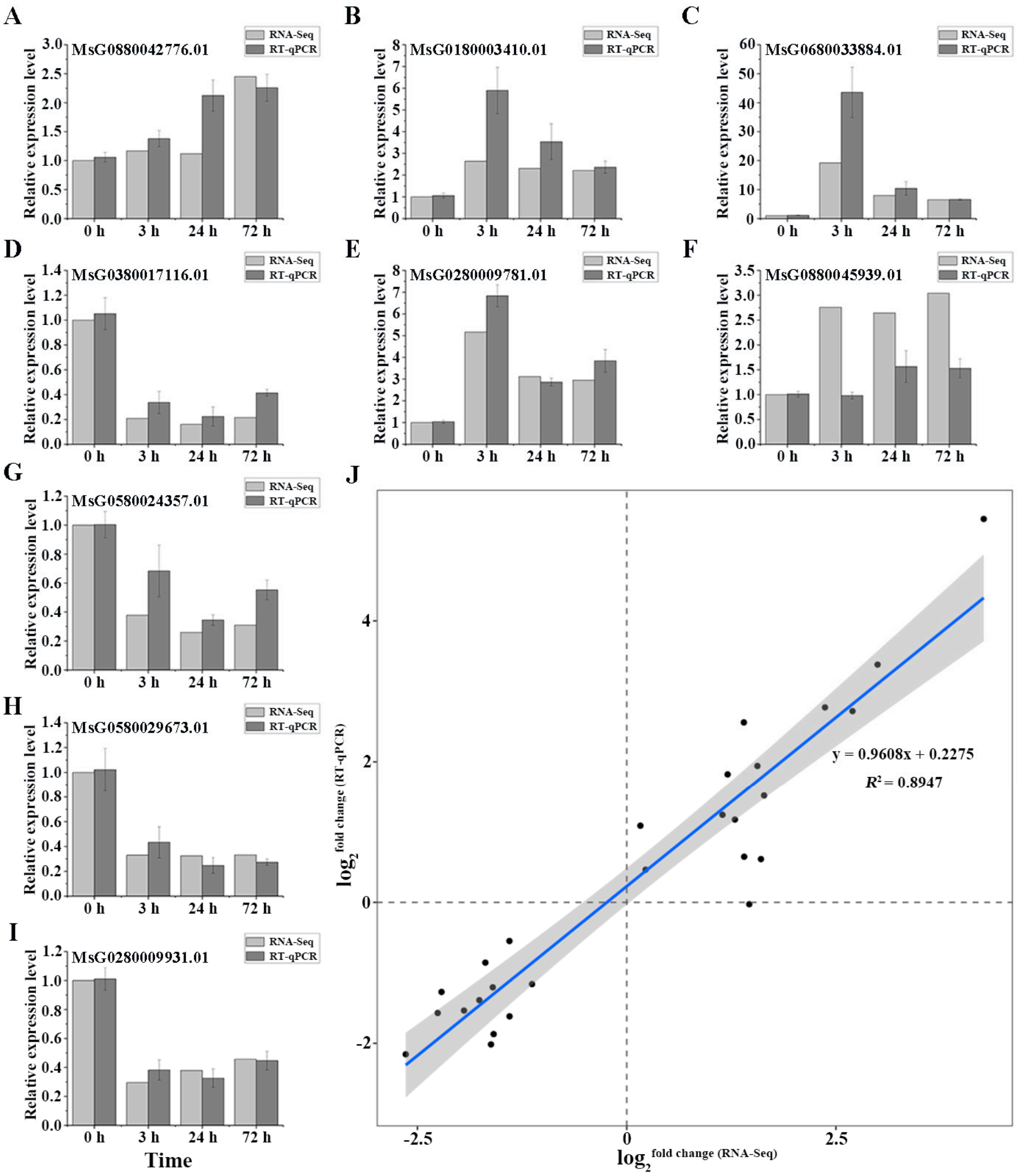

Figure S2. Expression profiles of nine genes and the correlation between RNA-Seq data and RT-qPCR data. (A) MsG0880042776.01. (B) MsG0180003410.01. (C) MsG0680033884.01. (D) MsG0380017116.01. (E) MsG0280009781.01. (F) MsG0880045939.01. (G) MsG0580024357.01. (H) MsG0580029673.01. (I) MsG0280009931.01. The RNA-Seq (fold change) and RT-qPCR (relative expression) data of nine genes were plotted using Origin. (J) The correlation between RNA-Seq data and RT-qPCR validation of nine genes.

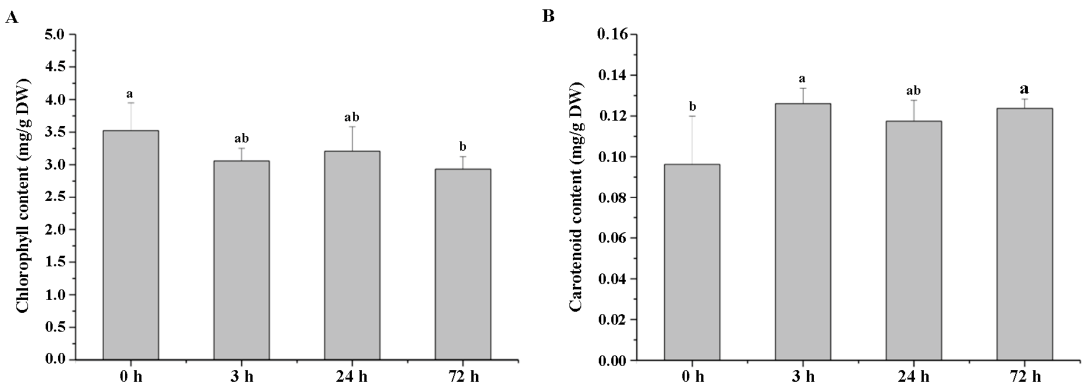

Figure S3. Chlorophyll and carotenoid contents at different time points under salt stress. (A) Chlorophyll content. (B) Carotenoid content. Data are presented as means ± SD of three independent biological replicates. Statistical significance was determined by one-way ANOVA followed by Fisher's LSD post hoc test. Different lowercase letters indicate statistically significant differences between groups ($p < 0.05$).

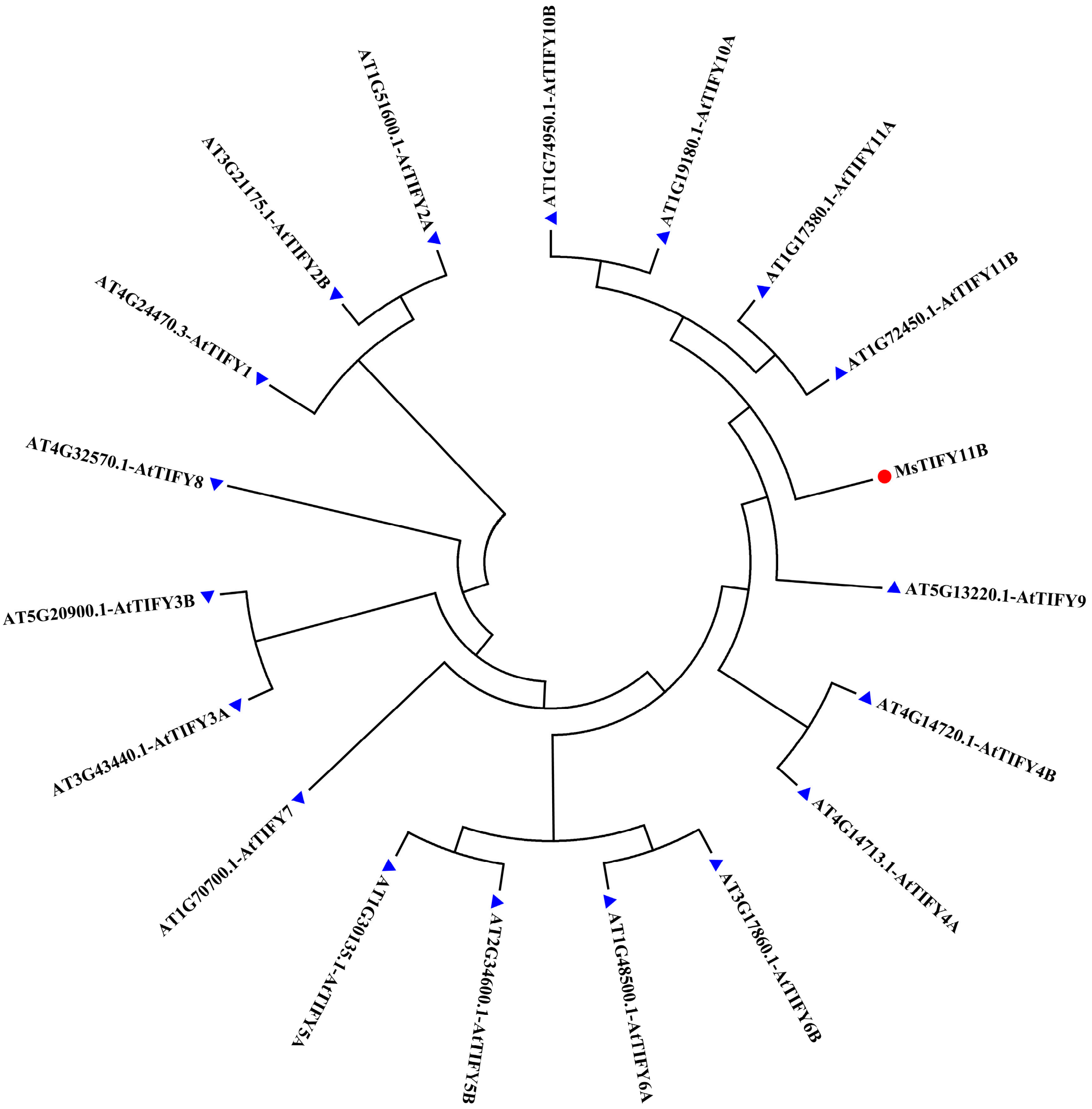

Figure S4. Phylogenetic analysis of MsTIFY11B and 18 Arabidopsis TIFY family members. Multiple sequence alignment was performed on 19 TIFY proteins, and a phylogenetic tree was constructed using MEGA7 with the neighbor-joining method and 1000 bootstrap replicates.
